# Long‐term outcomes of ventricular tachycardia ablation in repaired tetralogy of Fallot: Systematic review and meta‐analysis

**DOI:** 10.1002/joa3.13095

**Published:** 2024-06-12

**Authors:** Gusti Ngurah Prana Jagannatha, Brian Mendel, Nikita Pratama Toding Labi, Wingga Chrisna Aji, Anastasya Maria Kosasih, Jonathan Adrian, Bryan Gervais de Liyis, Putu Febry Krisna Pertiwi, I Made Putra Swi Antara

**Affiliations:** ^1^ Faculty of Medicine Udayana University, Prof. dr. I.G.N.G Ngoerah General Hospital Denpasar Bali Indonesia; ^2^ Department of Cardiology and Vascular Medicine Sultan Sulaiman Government Hospital Serdang Bedagai North Sumatra Indonesia; ^3^ Faculty of Medicine Sam Ratulangi University Manado North Sulawesi Indonesia; ^4^ Faculty of Medicine Muhammadiyah Yogyakarta University Yogyakarta Indonesia; ^5^ Division of Electrophysiology and Cardiac Pacing, Department of Cardiology and Vascular Medicine, Faculty of Medicine Udayana University, Prof. dr. I.G.N.G Ngoerah General Hospital Denpasar Bali Indonesia

**Keywords:** ablation, slow‐conducting anatomical isthmus, sudden cardiac death, tetralogy of Fallot, ventricular tachycardia

## Abstract

**Background:**

Ventricular tachycardia (VT) remains a risk in repaired Tetralogy of Fallot (rTOF); however, long‐term benefits of VT ablation have not been established. This study compares the outcomes of rTOF patients with and without VT ablation.

**Methods:**

We searched multiple databases examining the outcomes of rTOF patients who had undergone VT ablation compared to those without ablation. Primary outcomes were VT recurrence, sudden cardiac death (SCD), and all‐cause mortality. Subgroup analysis was conducted based on the type of ablation (catheter and surgical). Slow‐conducting anatomical isthmus (SCAI)‐based catheter ablation (CA) was also analyzed separately. The secondary outcome was the risk factors for the pre‐ablation history of VT.

**Results:**

Fifteen cohort studies with 1459 patients were included, 21.4% exhibited VTs. SCAI was found in 30.4% of the population, with 3.7% of non‐inducible VT. Factors significantly associated with VT before ablation included a history of ventriculostomy, QRS duration ≥180 ms, fragmented QRS, moderate to severe pulmonary regurgitation, high premature ventricular contractions burden, late gadolinium enhancement, and SCAI. Ablation was only beneficial in reducing VTs recurrence in SCAI‐based CA (risk ratio (RR) 0.11; 95% CI 0.03 to 0.33. *p* < 0.001; I^2^ = 0%) with no recurrence in patients with preventive ablation (mean follow‐up time 91.14 ± 77.81 months). The outcomes of VT ablation indicated a favorable trend concerning SCD and all‐cause mortality (RR 0.49 and 0.44, respectively); however, they were statistically insignificant.

**Conclusions:**

SCAI‐based CA has significant advantages in reducing VT recurrence in rTOF patients. Risk stratification plays a key role in determining the decision to perform ablation.

## Graphical abstract

Factors associated with a history of ventricular tachycardia before ablation and long‐term outcomes of ablation in rTOF patients.

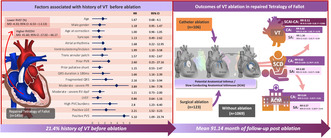

## 1 INTRODUCTION

The overall survival of tetralogy of Fallot (TOF) has improved dramatically since the Blalock–Taussig–Thomas shunt was first performed, initially as a palliative procedure. With more advanced surgical techniques becoming common practice, recent studies reveal that the majority of patients with repaired TOF (rTOF) survived into adulthood.^1^, ^2^, ^3^ However, the peri‐procedural and long‐term risks of arrhythmias, including ventricular tachycardia (VT) as well as sudden cardiac death (SCD), remain major concerns.^4^, ^5^, ^6^ Since the increased risk for postsurgical VTs was initially recognized, several risk factors and models have been proposed to help identify individuals most vulnerable to developing VTs.^7^ However, risk classification for patients with rTOF remains a challenge. Bayesian approach in stratifying the risk of rTOF patients to determine which group might benefit from performing an invasive electrophysiological study (EPS).^4^ This approach considers probabilistic risk prediction models. Unfortunately, further research examining the proposed parameters in a large rTOF population has not been identified.

The available evidence underscores that implantable cardioverter‐defibrillator (ICD) alone are deemed less preferable for patients with rTOF, as they do not possess inherent preventive capabilities against VTs. Nevertheless, ICDs demonstrate efficacy in terminating VT episodes, consequently lowering the susceptibility to SCD.^8^ Additionally, ICD implementation entails associated complications, including a notable incidence of inappropriate shocks, potential lead dysfunction, and an overarching detriment to the patient's overall quality of life.^8^, ^9^, ^10^, ^11^ The American Heart Association recommends ablation for the secondary prevention of VTs, and further supports the rationale of carrying out the procedure during the initial pulmonary valve replacement (PVR), specifically in patients with slow‐conducting anatomical isthmus (SCAI).^12^ Nonetheless, a significant knowledge gap becomes evident concerning the enduring recurrence trends and occurrences of SCD after VT ablation for rTOF.

The principal objective of this study is to compare the outcomes among patients with rTOF who underwent VT ablation with those who refrained from such intervention. Additionally, the study explores the discernible risk factors underpinning the development of VT in rTOF patients before the ablation procedure.

## 2 METHODS

This meta‐analysis was performed according to the Cochrane Collaboration and Preferred Reporting Items for Systematic Review and Meta‐Analyses (PRISMA)^13^

### 2.1 Search strategy

MEDLINE (Medical Literature Analysis and Retrieval System Online), including PubMed, EMBASE (Excerpta Medical Database), and Cochrane Library were examined from the earliest available evidence until September 7, 2023, using the following search string: “Tetralogy of Fallot” and (“Ablation” or “Resection” or “Isthmus” or “Arrhythmia”). The reference lists of the identified articles were also reviewed to determine additional citations.

### 2.2 Eligibility criteria

The following patient characteristics were taken into consideration when selecting studies: (1) studies reporting rTOF patient data who underwent catheter ablation (CA) and/or surgical ablation (SA) of sustained monomorphic VT (SMVT) and (2) studies reporting clinical outcomes of VT recurrence and/or SCD and/or all‐cause mortality of VT ablation compared to the non‐ablation group. The non‐ablation group included patients who did not undergo VT ablation regardless of the cause, including those who only underwent ICD implantation, exclusively sought anti‐arrhythmic drug (AAD) treatment, non‐inducible VT, absence of SCAI, or experienced ablation failure. Abstracts, case reports, conference presentations, editorials, reviews, and expert opinions were excluded from our analysis.

### 2.3 Primary and secondary outcomes

The primary outcomes of this study were VT recurrence, SCD, and all‐cause mortality during the follow‐up period after ablation. The secondary outcome was the risk factors for pre‐ablation VT. VT recurrence was defined as the occurrence of SMVT following ablation documented during the follow‐up period, whether documented on the ICD as demonstrated by the delivery of an appropriate shock or anti‐tachycardia pacing or documented on the 12‐lead ECG. All‐cause mortality was defined as the death of a patient from any or no stated cause in the follow‐up period after VT ablation. SCD was defined as an aborted or non‐aborted cardiac arrest following the ablation of VTs. History of VT was defined as the occurrence of clinical SMVT or inducible SMVT during an EPS before ablation.

### 2.4 Data extractions and quality appraisal

Four researchers conducted a comprehensive screening process, encompassing both titles and abstracts of potential studies. Disagreements were resolved through consensus, facilitated by the participation of a fifth referee. We gathered data about the characteristics of pre‐ablated rTOF patients who experienced VTs and those who did not. Extracted subjects in this study were the only subjects that mentioned the presence or absence of their outcomes that correspond with our primary and secondary outcomes. Special consideration was appointed to studies pertaining to the identical cohort, wherein data amalgamation was exclusively conducted when disparities existed in the outcomes. In scenarios where discrepant results were presented in both papers concerning the same outcomes, data from the more recent publication were opted for selection.

### 2.5 Quality assessment and statistical analysis

Studies qualities were assessed using the Newcastle‐Ottawa scale.^14^ Variables were only synthesized if at least two studies reported particular outcomes within the scope of this study. Continuous variables were presented as their mean and standard deviation (SD). The sample size, median, range, and quartiles were used to approximate the mean and SD if they were not reported^15^, ^16^ Heterogeneity between study populations was calculated using the *I* ^2^ statistic^17^ in which values less than or equal to 25%, 50%, and 75% are considered evidence of low, medium, and high‐level heterogeneity, respectively. Data were summarized across groups using the Mantel–Haenszel (M–H) risk ratio (RR) fixed effect model if *I* ^2^ < 25%. The random effect model was used if *I* ^2^ ≥ 25%.^18^ Funnel plots were used to evaluate publication bias.^18^, ^19^ Our analyses used 95% confidence intervals (95% CI) and were carried out using Review Manager 5.4.

## 3 RESULTS

**FIGURE 1:**
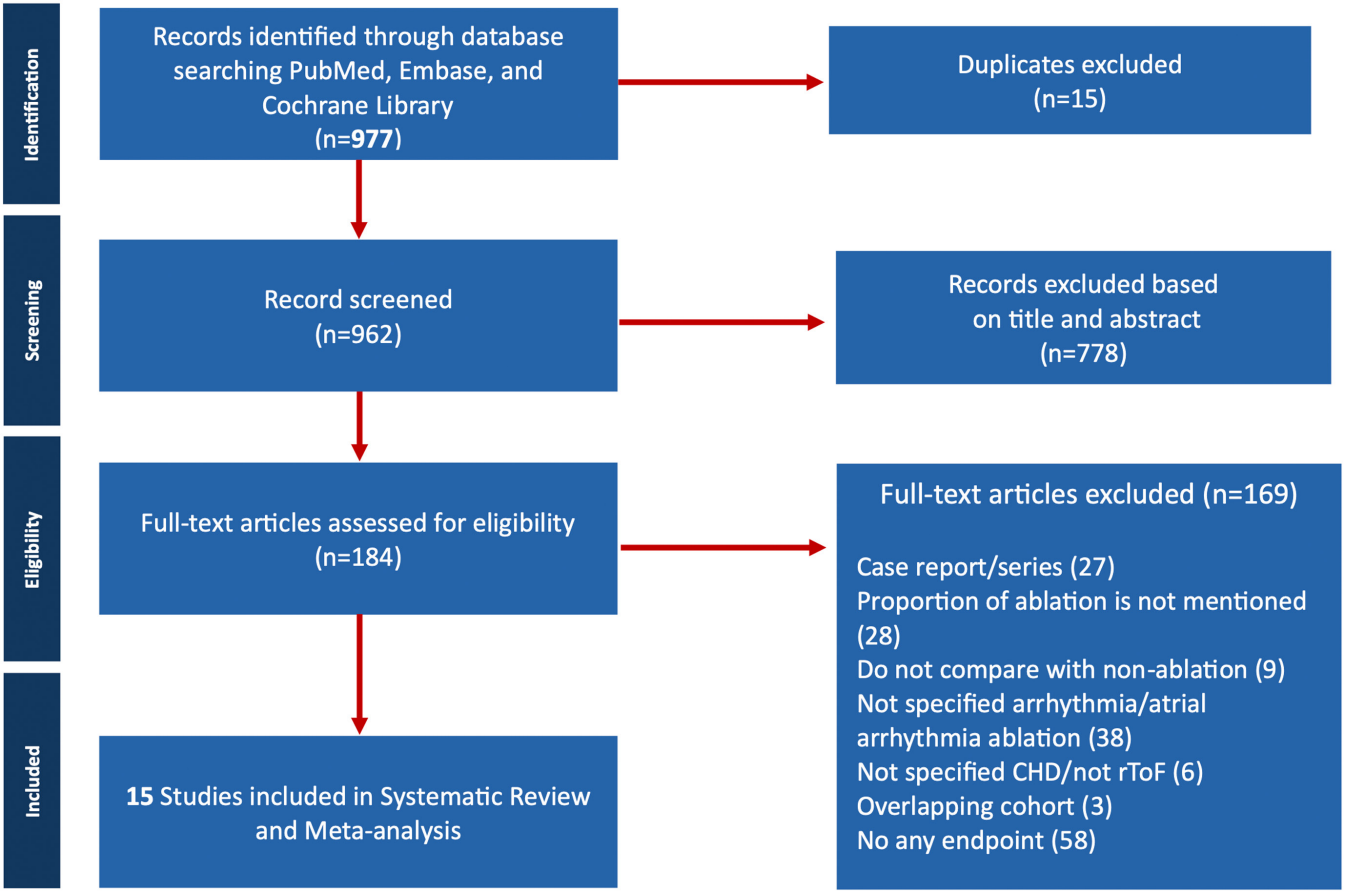
Preferred reporting items for systematic reviews and meta‐analyses (PRISMA) flow diagram. Depiction of selection of studies. CHD, congenital heart disease; rTOF, repaired Tetralogy of Fallot.

The PRISMA flow diagram in Figure 1 summarizes the study selection process. The initial search yielded a total of 977 studies. Considering additional searches (MEDLINE updates, reference lists), 184 potentially relevant studies were selected for full‐text review. All in all, 15 cohort studies were included in our data synthesis (Figure 1). In one of the 15 studies, particularly Kawada et al.^20^ was split into two sub‐studies for analysis as it included distinct data sets for CA and SA. Two studies by Kapel et al.^21^, ^22^ originated from the same cohort; thus, the synthesis of novel data from the most recent publication was prioritized and referred only to the earlier publication for variables absent in the more recent one.

### 3.1 Characteristics of included studies

Outcomes of CA^20^, ^21^, ^23^, ^24^, ^25^, ^26^, ^27^, ^28^ surgical cryoablation^20^, ^29^, ^30^, ^31^, ^32^, ^33^, ^34^, ^35^, ^36^ surgical radiofrequency ablation,^29^ and the resection combination^31^, ^35^ in rTOF patients with a history of VT or a high‐risk of VT were included in our meta‐analysis (Table 1). Some studies performed intraoperative SA or CA during late reoperation or PVR.^20^, ^26^, ^28^, ^29^, ^31^, ^32^, ^33^, ^34^, ^35^ Five studies^21^, ^22^, ^27^, ^28^, ^29^ specifically conducted SCAI‐based CA, with a generally similar definition of SCAI as an anatomical isthmus with conduction velocity <0.50 m/s. One study^22^ used the conduction velocity index as the reference, and another study^21^ added low bipolar voltage electrograms (<1.5 mV) as a second criterion for SCAI. Almost all studies included VT ablation outcomes as secondary data, except for the study by Kawada et al.^20^ The main objectives of these studies were to determine the risk factors for VTs, EPS prior to PVR, outcomes of PVR, outcomes of ICD implantation, and the inducibility of VTs in rTOF patients. Even though the initial study had a large population, only data corresponding to our study's primary outcomes were synthesized. This explains why our population data, as presented in Table 1, did not show an entirely cumulative pattern.

**TABLE 1:** Baseline characteristics of included studies.

No	First author, year	Study design	Baseline characteristics										
			Total population, *n*	History of VT, *n*	Ablated, *n*	Not ablated, *n*	Mean age	Mean age at total repair	Adjuvant AADs after repair	Preserved LV function (%)	Preserved RV function (%)	ICD *n* (%)	Mean follow‐up time (month)
1	Bessière, 2021^23^	Cohort Retrospective and Prospective. Multicenter	26	15	5	21	42.2	8.6	Beta‐blockers (*n* = 52). Combination of beta‐blockers and amiodarone (*n* = 22). amiodarone (*n* = 12). and other antiarrhythmics (*n* = 26)	NA	NA	26 (100)	82.8
2	Bouyer, 2023^28^	Cohort Retrospective. Single Center	77	18	28	49	36.2	4.4	NA	NA	NA	5 (6.4)	74
3	Chiu, 2017^29^	Cohort Prospective. Single Center	12	12	6	6	27.1	4.4	Beta‐blockers (*n* = 4). amiodarone (*n* = 1)	NA	NA	12 (100)	306
4	Ghai, 2002^30^	Cohort Retrospective. Single Center	137	12	3	134	46.25	5	Amiodarone (*n* = 4). propafenone (*n* = 1). sotalol (*n* = 1). digoxin (*n* = 3)	NA	NA	2 (1.4)	252
5	Harisson, 1997^31^	Cohort Retrospective. Single Center	210	18	10	8	33.2	11.2	Amiodarone (*n* = 13). mexiletine (*n* = 9). quinidine (*n* = 7). sotalol (*n* = 7). procainamide (*n* = 4) and propafenone (*n* = 4)	NA	NA	1 (0.4)	48
6	Kapel, 2017^22^	Cohort Retrospective. Multi Center	74	28	19	55	40	5.9	Not specified (*n* = 3)	46 (100)	28 (61)	18 (24.3)	50
7	Kapel, 2018^21^	Cohort Retrospective. Multi Center	78	24	16	62	37	5.0	Class III anti‐arrhythmic drugs (*n* = 2)	76 (97)	47 (60)	NA	39
8	Karamlou, 2005^36^	Cohort Retrospective. Single Center	249	44	31	218	26.9	7.95	NA	NA	NA	NA	90
9	Kawada, 2021^20^	Cohort Retrospective. Single Center	47	33	20	27	43.1	8.5	Beta blocker (*n* = 23) and amiodarone (*n* = 18)	NA	NA	47 (100)	93.9
10	Kimura, 2023^27^	Cohort Retrisoective	48	14	17	31	34	2.6	NA	48 (100)	42 (87)	3 (6.2)	24.8
11	Rotes, 2014^32^	Cohort Retrospective. Single Center	205	19	22	183	33.1	6.2	NA	154 (81.4)	152 (86.8)	15 (7.3)	80.4
12	Sandhu, 2018^33^	Cohort Prospective. Multicenter	70	34	31	39	34.8	4	Class III antiarrhythmic drugs (*n* = 4) and beta‐blocker (*n* = 24)	NA	15 (21.5)	14 (20)	73.2
13	Therrien, 2001^35^	Cohort Prospective. Multicenter	70	15	9	61	27.86	9.76	Not specified (*n* = 11)	NA	NA	NA	60
14	Waldmann, 2023^26^	Cohort Prospective. Multicenter	120	27	24	96	39.2	NA	NA	NA	NA	10 (8.3)	12.4
15	Warner, 2003^34^	Cohort Retrospective. Single Center	36	NA	2	34	15.2	3.2	NA	NA	NA	NA	80.6

No	First author, year	Specific patient characteristics/inclusion criteria	Description of procedures and outcomes			
			Type of ablation	Presence of SCAI	Preventive SCAI‐based ablation, *n* (%)	Definition of VT recurrency
1	Bessière. 2021^23^	rTOF undergoing PVR	Catheter ablation	NA	NA	Documented on ICD
2	Bouyer, 2023^28^	rTOF undergoing PVR	Catheter—Radiofrequency ablation or Surgical—Cryoablation	18/18 in inducible VT 11/59 in non‐inducible VT	11 (100)	Documented on 24 h Holter monitor recordings, ICD interrogations, or 12 lead ECG
3	Chiu. 2017^29^	rTOF undergoing EP study	Surgical—Cryo or radiofrequency ablation	NA	NA	Documented on ICD
4	Ghai. 2002^30^	Adult rTOF (>18 years old) with SCD and non‐SCD	Surgical—Cryoablation	NA	NA	Documented clinically
5	Harisson. 1997^31^	rTOF undergoing PVR	Surgical Cryoablation ± Resection in adjuvant to second operation	NA	NA	Documented clinically
6	Kapel. 2017^22^	Clinically Documented VT after TOF Repair	Catheter—Radiofrequency ablation	26/28 in inducible VT 2/46 in non‐inducible VT	2 (100)	Documented on ICD
7	Kapel. 2018^21^	rTOF with QRS duration ≥180 ms. Nonsustained VT on Holter. at least moderate dysfunction of the LV or RV. late repair (≥5 years of age) and the presence of a transannular patch	Catheter—Radiofrequency ablation	22/24 in inducible VT 2/54 in non‐inducible VT	2 (100)	Documented on ICD or clinically
8	Karamlou. 2005^36^	rTOF with either documented sustained VT. or considered at risk for VT and/or with indication for reoperation who underwent electrophysiological evaluation and RV electroanatomical mapping	Surgical—Cryoablation	NA	NA	Documented on 12‐lead ECG. Holter recording. or ECG strips
9	Kawada. 2021^20^	rTOF who underwent initial ICD/CRT‐D placement	Catheter—Radiofrequency ablation and Surgical—Cryoablation	NA	NA	Documented on ICD
10	Kimura, 2023^27^	rTOF who underwent 3D‐EAM and 3D LGE‐CMR	Catheter—Radiofrequency ablation	20/48	NA	Documented on ICD
11	Rotes. 2014^32^	rTOF who underwent reoperation to restore pulmonary valve function	Surgical—Cryoablation	NA	NA	Documented on a 12‐lead ECG. Holter recording. or ECG strips
12	Sandhu. 2018^33^	rTOF patients who underwent EPS at time of PVR	Surgical—Cryoablation	NA	NA	In the ablation group as documented on ICD and clinically
13	Therrien. 2001^35^	rTOF undergoing surgical PVR and concomitant EP study	Surgical—Cryoablation	NA	NA	Documented on clinically
14	Waldmann, 2023^26^	rTOF patients who underwent EPS at time of PVR	Catheter—Radiofrequency ablation	33/120	6 (100)	Documented on ICD
15	Warner. 2003^34^	rTOF undergoing PVR because of progressive pulmonary regurgitation	Surgical—Cryoablation and Endocardial Resection	NA	NA	Documented on clinically

Abbreviation: 3D‐EAM, 3D‐electroanatomical mapping; AADs, anti‐arrhythmic drugs; CRT‐D, cardiac resynchronization therapy defibrillator; EP, electrophysiology; ICD, implantable cardiac defibrillator; LV, left ventricle; PVR, pulmonary valve replacement; RV, right ventricle; SCAI, slow‐conducting anatomical isthmus; SCD, sudden cardiac death; TOF, tetralogy of Fallot; VT, ventricular tachycardia.

### 3.2 Risk of bias

The overall quality of the included studies was in the category of high‐quality or satisfactory studies, with NOS scores varying from 5 to 7 (Table S1). One potential risk of bias presents in the fact that most studies report on ablation outcomes as secondary data, with the study by Kawada et al. being an exception^20^ Three studies are categorized as satisfactory^21^, ^22^, ^31^ Compared to other studies, we considered these studies to have insufficient follow‐up duration (below 5 years), which we deemed insufficient to observe a meaningful result in VT recurrence.

### 3.3 Data synthesis

**FIGURE 2:**
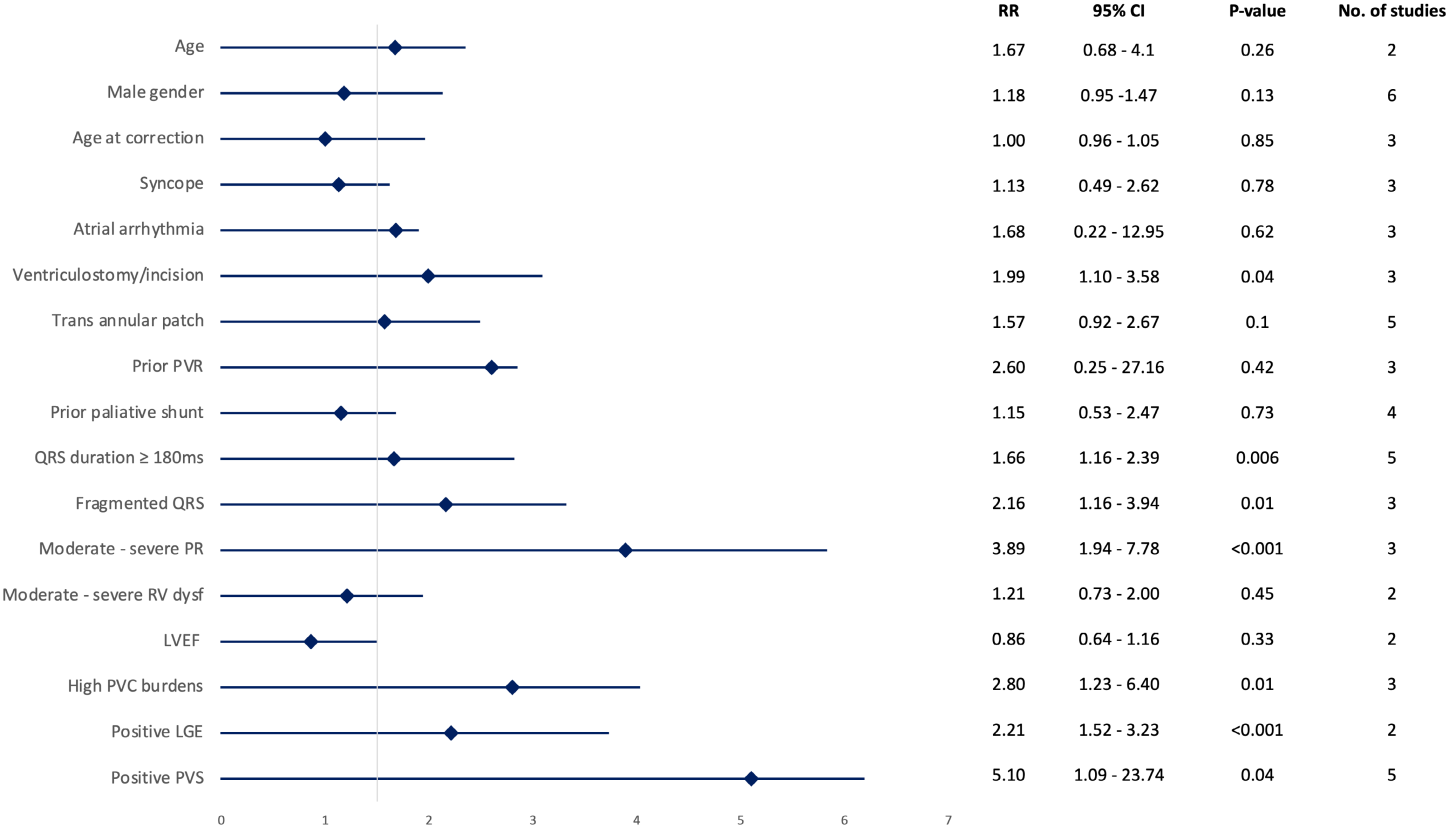
Forest plot risk ratio of risk factor associated with history of VT in rTOF. Dysf, dysfunction; LGE, late gadolinium enhancement; LVEF, left ventricular ejection fraction; NSVT, non‐sustain ventricular tachycardia; PR, pulmonary regurgitation; PVR, pulmonary valve replacement; PVS, programmed ventricular stimulation; RV, right ventricle.

A cumulative total of 1459 patients were encompassed across the 15 cohorts that were incorporated into the analysis. Out of those involved, 313 patients had a history of VTs that were clinically documented or inducible during EPS before receiving ablation, and a further 229 underwent VT ablation. From a total of 397 patients who underwent 3D‐Electroanatomical mapping (3D‐EAM), 121 patients had SCAI with an overall percentage of 3.7% SCAI without inducible VT before receiving ablation. All SCAIs in the SCAI‐based CA group achieved acute success, which is defined as the achievement of bidirectional block and non‐inducibility of VT during the procedure. The total mean age was 34.4 ± 7.5 years, and the mean age at TOF repair was 6.1 ± 2.5 years. ICD was implanted in at least 10.2% of the patients. In terms of demographic characteristics (Figure 2).

**FIGURE 3:**
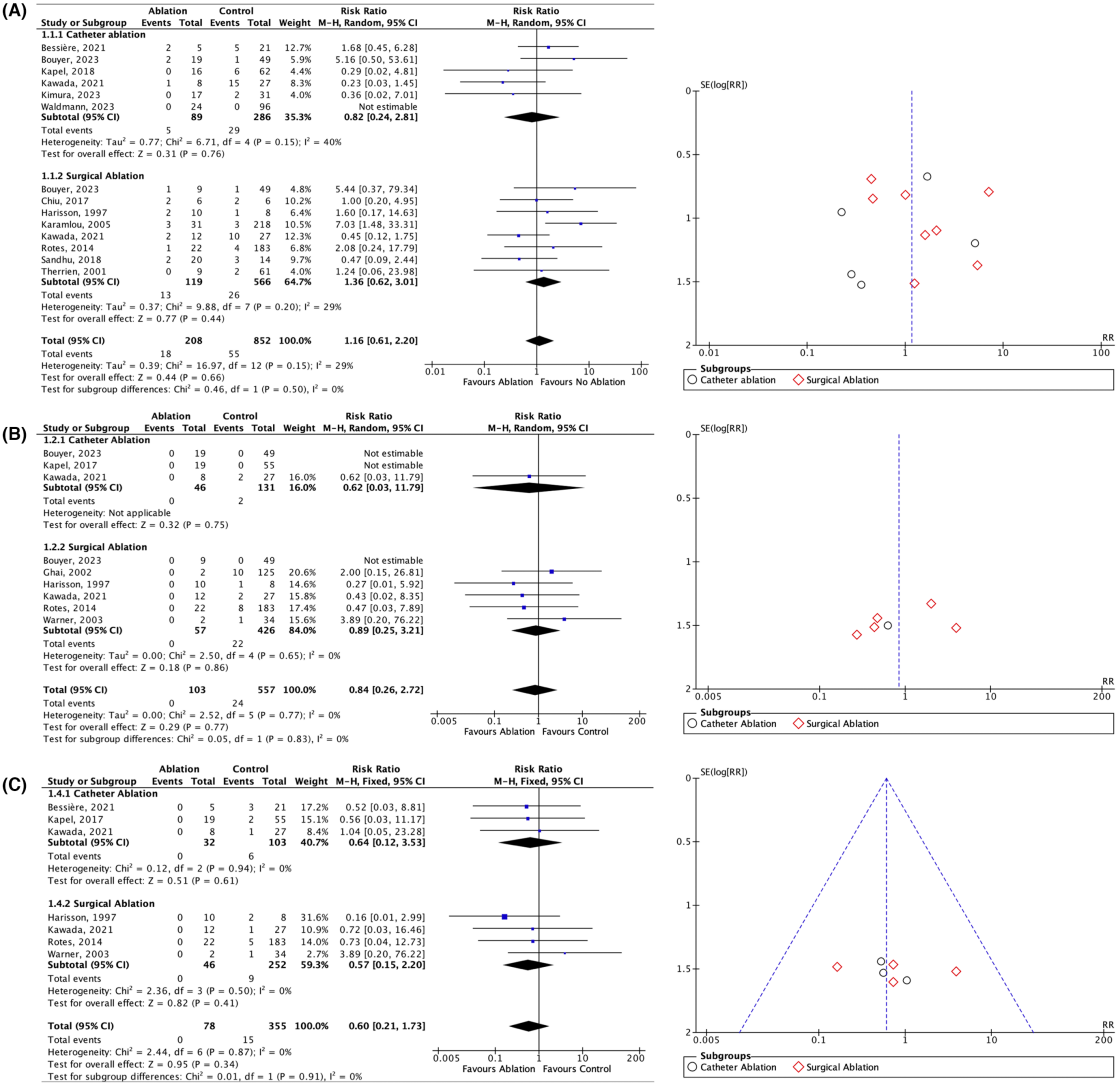
Forest plot risk ratio with random effect models or fixed effect models of clinical outcomes of VT ablation in rTOF, (A) VT recurrency, (B) SCD, (C) All‐cause mortality. SCD, sudden cardiac death; VT, ventricular tachycardia; M–H, Mantel–Haenszel, CI, confidence interval.

**FIGURE 4:**
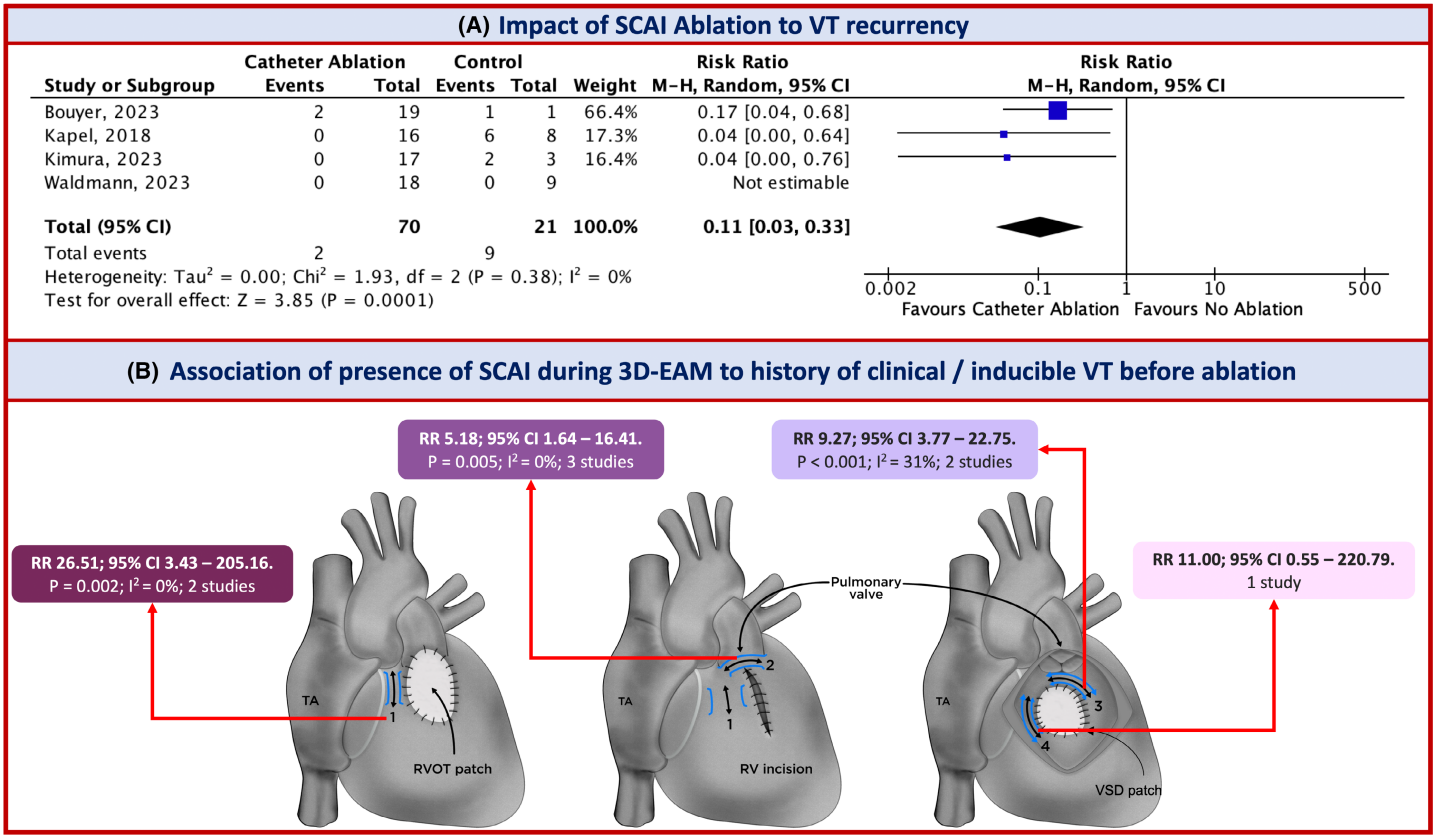
(A) Forest plot risk ratio with random effect models of impact of SCAI ablation to VT recurrency in rTOF, (B) Summary of risk ratio with random effect models analysis by Review Manager of association of the presence of SCAI during 3D‐EAM to the history of clinical or inducible VT before ablation. 3D‐EAM, 3D‐Electroanatomical mapping; SCAI, slow‐conducting anatomical isthmus; VT, ventricular tachycardia; M–H, Mantel–Haenszel, CI, confidence interval, SE (log[RR]), Standard error (log[risk ratio]).

In the primary outcome after a mean follow‐up of 91.14 ± 77.81 months, there was a trend toward decreasing the risk of VT recurrence in the CA group [RR 0.82]. Conversely, there was an increasing risk in the SA subgroup [RR 1.36], although this was not statistically significant (Figure 3A). However, SCAI‐based CA (with a mean follow‐up of 40.04 ± 21.21 months) was beneficially proven to decrease VT recurrence approximately 10‐fold [RR 0.11; 95% CI 0.03 to 0.33. *p* < 0.001; I2 = 0%] (Figure 4A). All patients with SCAI without induced VT were also ablated, and no recurrence was found during follow‐up. CA and SA demonstrated a positive outcome trend for SCD and all‐cause mortality (RR 0.84 and 0.60, respectively), but they did not reach statistical significance (Figure 3B,C). Furthermore, our funnel plots did not suggest significant publication bias for comparing VT recurrence, SCD, and all‐cause mortality in all groups (Figures S1–S3).

Regarding the secondary outcome of risk factors associated with the first episode of VT after TOF repair and before the first ablation, it was found that male gender and age were non‐significantly associated with developing VTs in the rTOF population. Regarding surgical history, rTOF with subsequent VT was significantly associated with a history of ventriculostomy/ventricular incision [RR 1.99; 95% CI 1.10 to 3.58. *p* = 0.04; *I* ^2^ = 0%]. Other factors associated with the incidence of VTs in rTOF patients included QRS duration ≥180 ms [RR 1.66; 95% CI 1.16 to 2.39. *p* = 0.006; *I* ^2^ = 0%], presence of fragmented QRS [RR 2.16; 95% CI 1.16 to 3.94. *p* = 0.01; *I* ^2^ = 4%], high premature ventricular contraction (PVC) burdens or Non‐sustained VT (NSVT) during the 24‐h Holter monitoring [RR 2.80; 95% CI 1.23 – 6.40. *p* = 0.01; *I* ^2^ = 0%], moderate to severe pulmonary regurgitation (PR) [RR 3.89; 95% CI 1.94 to 7.78. *p* < 0.001; *I* ^2^ = 0%], and late gadolinium enhancement (LGE) in cardiovascular magnetic resonance (CMR) [RR 2.21; 95% CI 1.52 to 3.23. *p* < 0.001; *I* ^2^ = 0%]. Based on CMR anatomical parameters, patients who experienced VT had a lower right ventricular ejection fraction (RVEF) and higher right ventricular end‐diastolic volume index (RVEDVi) [MD −4.33; 95% CI −6.53 to −2.13; *p* < 0.001; *I* ^2^ = 0% and MD 41.60; 95% CI −17.02 to 66.17; *p* < 0.001; *I* ^2^ = 56%, respectively]. Moreover, rTOF patients who had undergone EPS with the subsequent discovery of an inducible VT on programmed ventricular stimulation (PVS) had a fivefold increased risk of developing VTs [RR 5.10; 95% CI 1.09 to 23.74. *p* = 0.04; *I* ^2^ = 64%]. The presence of SCAI during 3D‐EAM had a sevenfold association with a history of VT by low heterogeneity [RR 7.76; 95% CI 4.67 to 19.91. *p* < 0.001; *I* ^2^ = 0%] and the history of VT based on subgroup analysis of SCAI types can be found in Figure 4B. Further information on forest plots and funnel plot analysis of risk factors associated with the development of VTs in pre‐ablation rTOF can be found in the Figures S4–S23.

## 4 DISCUSSION

The key findings of this meta‐analysis are as follows: (1) VT ablation in rTOF patients, particularly SCAI‐based CA, is beneficial in reducing VT recurrence. (2) The prevalence of VT in this population is 21.4%, despite TOF repair. (3) Several risk factors have been linked to an increased incidence of VTs prior to ablation in rTOF patients, including a history of ventriculostomy/ventricular incision during repair, QRS duration ≥180 ms, fragmented QRS, and moderate to severe PR. (4) Less invasive diagnostic techniques, including 24‐h Holter monitoring to detect NSVT or high PVC burden and CMR to determine LGE, RVEF, and RVEDVi may be helpful in stratifying patients based on risk factors before recommending more invasive diagnostic techniques. (5) Finally, we found VT inducibility and SCAI findings during EPS associated with VT, which can guide the decision to perform ablation. In our collective understanding, this study represents the first meta‐analysis assessing the long‐term outcomes of VT ablation in patients with rTOF.

Ventricular arrhythmias (VA) remain a risk in rTOF patients throughout adulthood, and the true prevalence of VA is unknown, SCD in rTOF patients is believed to be mainly provoked by VAs.^12^ Although ICD implantation has been shown to be beneficial in the prevention of SCD, long‐term follow‐up of ICD implantation outcomes in >4000 adults with congenital heart disease (CHD), predominantly rTOF, reported a high incidence of complications, with more than a third suffering from severe complications (9% per year). This study concluded that the risks and benefits of ICD implantation depend on the patient and the disease and should be clearly discussed prior to implantation.^11^ The predominant form of VA in rTOF is SMVT, which is the underlying cause for the majority of ICD treatment (>80%).^37^ According to a previous meta‐analysis, the results of substrate‐based CA in patients with structural heart disease, including rTOF, are extremely encouraging because of the low rate of VT recurrence.^38^ The same outcomes were seen in our investigation. The promising outcome of ablation in rTOF has been highlighted previously by Groot et al. in atrial arrhythmia ablation as it is associated with predictable re‐entry circuits because of surgical repair procedures.^39^ Likewise, VT in rTOF occurs within a re‐entry in a predictable circuit that crosses clearly defined anatomical isthmus.^12^ Electrical barrier regions, including patch material, valve annuli, and dense postoperative scars, are retained in rTOF.^40^ Conduction between these barriers can slow down with time, allowing re‐entry around these electrical barriers.

The four identified anatomical isthmuses in rTOF are as follows: isthmus 1, which is located between the right ventricular outflow tract (RVOT) incision or patch and the tricuspid annulus; isthmus 2, between the RVOT incision and the pulmonary valve; isthmus 3, between the pulmonary valve and the ventricular septal defect patch; and isthmus 4, between the ventricular septal defect patch and the tricuspid annulus.^4^, ^21^, ^22^, ^40^, ^41^, ^42^, ^43^ A successful ablation achieves a bidirectional block by transmural lesion, minimizing the possibility of recurrence. In order to determine this with accuracy, operators may perform voltage mapping to measure the depth of ablation, which is routinely performed in CA but not in SA.^36^ As is the case, incompletely lesioned anatomical isthmus may not achieve the desired bidirectional block, promoting new slow‐conducting substrates that increase the risk of re‐entry.^22^ In line with the theory, we observed trend of increased recurrence with SA, although statistically insignificant. Another challenge of VT ablation in rTOF, if not using voltage mapping, is the presence of prosthetic material that can impede lesion formation, resulting in incomplete lesions or only achieving temporary non‐inducibility of VT.^44^ This is also supported by previous reports that noted the role of left‐sided SCAI‐based CA in addressing ablation challenges associated with myocardial hypertrophy and prosthetic materials in rTOF, which could potentially hinder complete ablation from the right side alone.^40^ Moreover, not every anatomical isthmus develops into a substrate for re‐entry. Isthmus width and the conduction velocity passing through it play an important role in determining re‐entry potential.^22^, ^45^ In rTOF patients with preserved ventricular function, precise 3D‐EAM during sinus rhythm revealed that only narrow and slow anatomical isthmus (conduction velocity <0.5 m/s) carried re‐entry potential for all reported and induced VTs.^22^ This information would only be available to operators when EPS with voltage mapping is performed. This rationale explains our analysis, which demonstrates an insignificant decreasing trend in VT recurrence in the general CA group and a significant decrease only observed in the SCAI‐based CA group.

Interestingly, our analysis demonstrated that 2.8% of rTOF patients with SCAI who underwent CA experienced VT recurrence. These findings suggest that substrate elimination does not completely rule out the possibility of recurrence. Several studies reporting on the characteristics of postablation VTs found that these arrhythmias had a different morphology and cycle length than the original VTs, indicating these may have been new VTs and not true recurrences.^20^, ^25^, ^29^ VT recurrence in SCAI‐based CA in our study was contributed by the study of Bouyer et al., all of whom had poor ventricular function.^28^ The one VT in the non‐ablation group in that study occurred in a patient without any risk factors. These findings suggest that other mechanisms may be responsible for the development of VT in rTOF than the aforementioned anatomical isthmus. In this study, we found that at least 19.7% of rTOF patients were reported to have an ICD implanted, although it was unclear what proportion of these patients had also been ablated. We believe that ICD serves as a potential confounder in our analysis since (1) ICDs terminate all forms of VTs, thus reducing mortality, (2) the data is unclear on the proportion of ICD placement in both ablated and non‐ablated patients, and (3) unclear proportion of patients with preserved ventricular function. We reason that this may be the rationale behind the statistical insignificance in SCD and all‐cause mortality between ablated and non‐ablated rTOF groups.^46^

**FIGURE 5:**
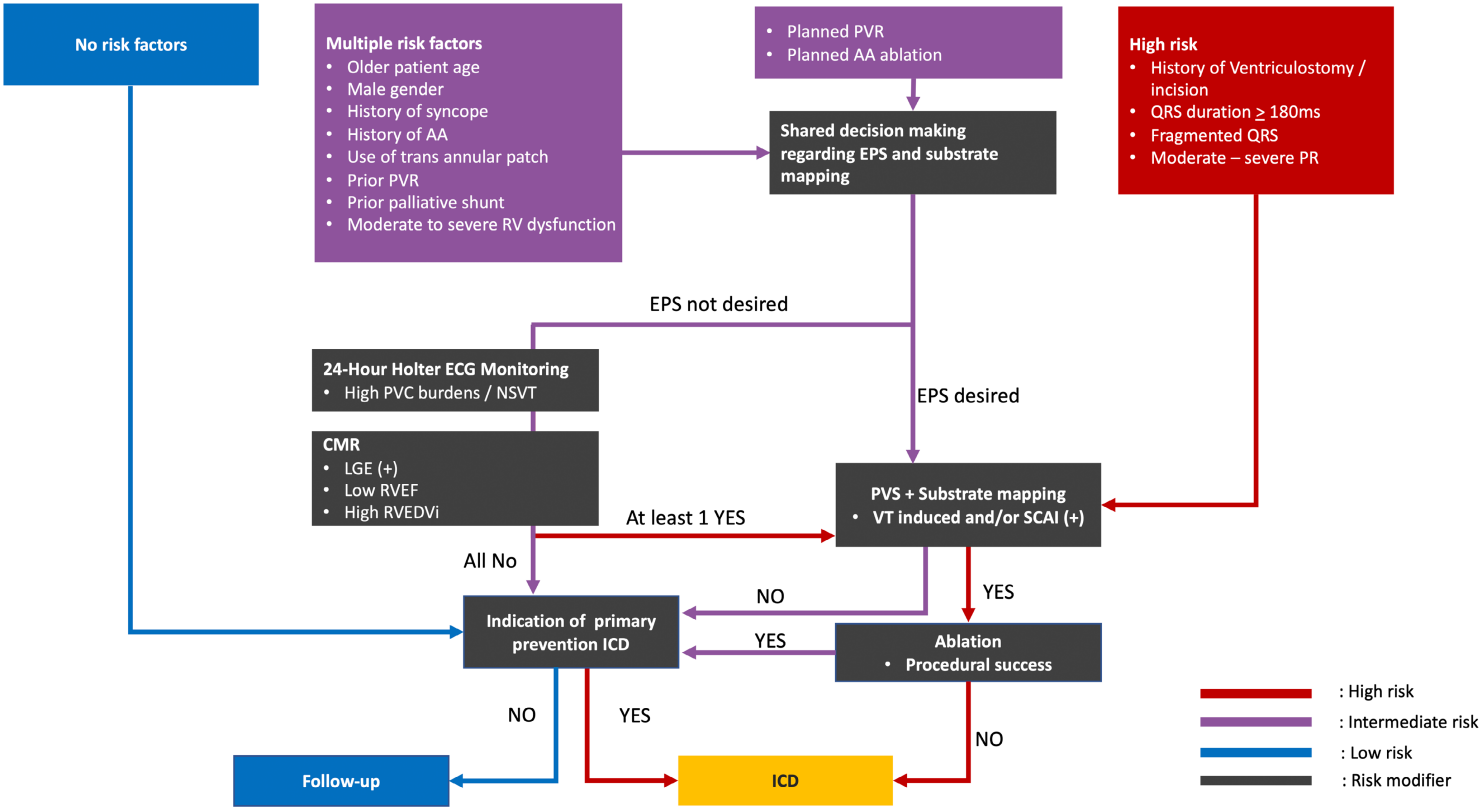
Proposed algorithm for the determination of invasive EPS, VT ablation, and ICD use. AA, atrial arrhythmia; CMR, cardiac magnetic resonance; ECG, electrocardiography; EPS, electrophysiology study; ICD, implantable cardiac defibrillator; PR, pulmonary regurgitation; PVC, premature ventricular contraction; PVR, pulmonary valve replacement; PVS, programmed ventricular stimulation; RV, right ventricular; RVEF, right ventricular ejection fraction; RVEDVi, Right Ventricular End‐diastolic volume index; SCAI, slowly conducting anatomical isthmuses; VT, ventricular tachycardia; other abbreviations as in Figure 2.

Through reflection on the Bayesian Approach for risk stratification in rTOF patients as proposed in Cohen et al.,^4^ we found that only several key variables (history of ventriculostomy/incision, QRS duration ≥180 ms, fragmented QRS, high PVC burden/NSVT on 24‐h Holter monitoring, moderate to severe PR, and LGE, RVEF, and RVEDVi on CMR and VT inducibility on EPS) are significantly associated with the incidence of pre‐ablation VTs. Moreover, other factors showed a trend toward increasing VT risk, although statistically insignificant. This may be because of the lack of studies considering these factors when investigating VTs. Because of these reasons, it is imperative not to overlook these factors when stratifying for VT risk. In our proposed algorithm (Figure 5), these factors are categorized as intermediate‐risk. In his paper, Cohen et al.^4^ add that the stratification approach would benefit from including patient demographics, clinical history, electrocardiographic findings, and ventricular dysfunction. According to these findings, we propose that patients with high‐risk features, as found statistically significant in analysis, should directly undergo EPS. In contrast, we also propose that patients without any risk factors, categorized as low‐risk for VT, continue annual surveillance. For patients with intermediate‐risk factors, we recommend considering non‐invasive testing to categorize their risk as high‐risk or low‐risk if the patient does not desire EPS. Our study found that several findings in non‐invasive diagnostic techniques (LGE, RVEF, and RVEDVi on CMR and high PVC burden/NSVT during 24‐h Holter monitoring) were significantly associated with an increased risk for VT. These techniques make excellent risk modifiers for patients categorized as an intermediate risk before deciding to escalate into more invasive techniques, such EPS. Finally, in addition to VT inducibility on PVS, the presence of SCAI during mapping should be considered when deciding on ablation. This idea is supported by several studies that ablate rTOF patients with SCAI in the absence of VT induction (preventive ablation) and report that residual SCAI is strongly associated with VT recurrence.^21^ Achievement of bidirectional block across the SCAI has been shown to result in 0% rates of VT recurrence.

## 5 STUDY LIMITATION

This study inherently exhibits the customary limitations inherent to systematic reviews and meta‐analyses. The entirety of the 15 studies within this meta‐analysis are observational, and while generally demonstrating commendable quality, they inherently possess a diminished statistical potency compared to expansive randomized controlled trials (RCTs). Furthermore, operational method such as transannular patch type RVOTR operation may affect the significant severity PR as the risk factors of VT in rTOF; therefore, further research is needed. The presence of an inadequate follow‐up duration further constrains the precise determination of recurrence proportions, given the potential necessity for the manifestation of such occurrences over extended periods spanning many years. Moreover, numerous data points were sourced from studies wherein they were initially examined as secondary data. This facet introduces an inherent susceptibility to bias stemming from plausible confounding factors.

## 6 CONCLUSIONS

SCAI‐based CA has significant advantages in reducing VT recurrence in rTOF patients with or without inducible VT, and CA generally demonstrated a trend in reducing SCD and all‐cause mortality. Based on secondary data analysis, we proposed an algorithm for VT risk stratification in rTOF patients before proceeding with EPS until the decision for ablation. Further large‐scale RCTs focusing on the long‐term outcomes of VT ablation and validating this risk stratification algorithm in rTOF are needed.

## CONFLICT OF INTEREST STATEMENT

The authors have no conflicts of interest to declare.

## ETHICS STATEMENT

No human participant was involved in this study.

## TRIAL REGISTRATION

This meta‐analysis was registered in the PROSPERO database with registration number CRD42023422892.

## Supporting information


Data S1.
