# Supplementary material for: Long‐term outcomes of ventricular tachycardia ablation in repaired tetralogy of Fallot: Systematic review and meta‐analysis
Source: J Arrhythm. 2024 Jun 12;40(4):935–47. doi: 10.1002/joa3.13095 (PMC11317698; doi:10.1002/joa3.13095)
Supplement: Supplementary file 1 — Data S1. [file JOA3-40-935-s001.docx]

**Supplementary Material**

**Supplementary Table 1: Quality Assessment**

| **Study** | **Selection** | | | | **Comparability** | | **Outcome** | | | **Total** |
| --- | --- | --- | --- | --- | --- | --- | --- | --- | --- | --- |
|  | **1** | **2** | **3** | **4** | **1** | **2** | **1** | **2** | **3** |  |
| Bessière, 2021 | $*$ | $*$ | $*$ | $*$ | **-** | **-** | $*$ | $*$ | $*$ | 7 |
| Bouyer, 2023 | $*$ | $*$ | $*$ | $*$ | **-** | **-** | $*$ | $*$ | $*$ | 7 |
| Chiu, 2017 | $*$ | $*$ | $*$ | $*$ | **-** | **-** | **-** | $*$ | $*$ | 6 |
| Ghai, 2002 | $*$ | $*$ | $*$ | $*$ | **-** | **-** | **-** | $*$ | $*$ | 6 |
| Harisson, 1997 | $*$ | $*$ | $*$ | $*$ | **-** | **-** | **-** | **-** | $*$ | 5 |
| Kapel, 2017 | $*$ | $*$ | $*$ | $*$ | **-** | **-** | **-** | **-** | $*$ | 5 |
| Kapel, 2018 | $*$ | $*$ | $*$ | $*$ | **-** | **-** | **-** | **-** | $*$ | 5 |
| Karamlou, 2005 | $*$ | $*$ | $*$ | $*$ | - | - | **-** | $*$ | $*$ | 6 |
| Kawada, 2021 | $*$ | $*$ | $*$ | $*$ | $*$ | - | **-** | $*$ | $*$ | 7 |
| Kimura, 2023 | $*$ | $*$ | $*$ | $*$ | **-** | **-** | **-** | $*$ | $*$ | 7 |
| Rotes, 2014 | $*$ | $*$ | $*$ | $*$ | **-** | **-** | **-** | $*$ | $*$ | 6 |
| Sandhu, 2018 | $*$ | $*$ | $*$ | $*$ | **-** | **-** | **-** | $*$ | $*$ | 6 |
| Therrien, 2001 | $*$ | $*$ | $*$ | $*$ | **-** | **-** | **-** | $*$ | $*$ | 6 |
| Waldmann, 2023 | $*$ | $*$ | $*$ | $*$ | **-** | **-** | $*$ | $*$ | $*$ | 7 |
| Warner, 2003 | $*$ | $*$ | $*$ | $*$ | **-** | **-** | **-** | $*$ | $*$ | 6 |

**Supplementary Figure 1.** Funnel plot VT recurrency of clinical outcomes of VT ablation in rTOF. SE(log[RR]) = Standard error (log[Risk ratio]).

**
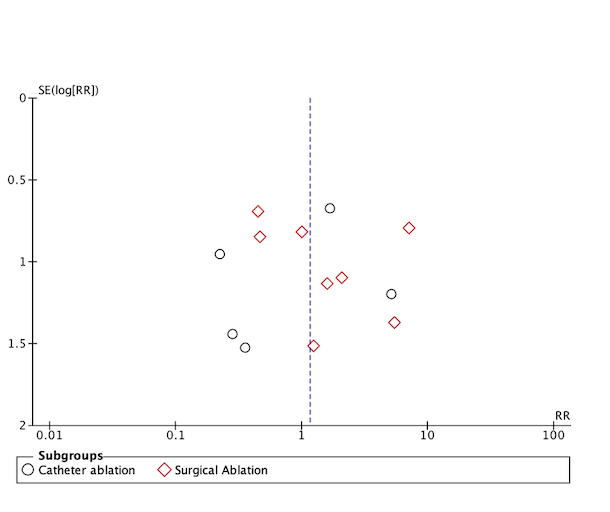
**

**Supplementary Figure 2. Funnel plot sudden cardiac death of clinical outcomes of VT ablation in rTOF. SE(log[RR]) = Standard error (log[Risk ratio]).**


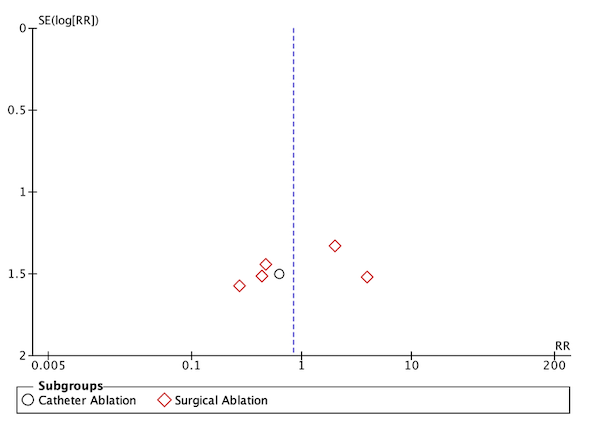


**Supplementary Figure 3. Funnel plot all-cause mortality of clinical outcomes of VT ablation in rTOF. SE(log[RR]) = Standard error (log[Risk ratio]).**


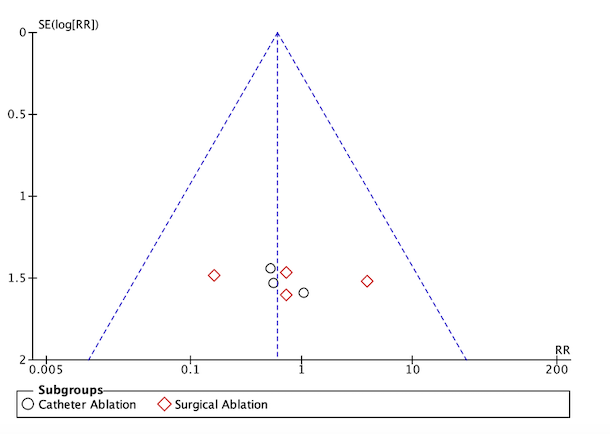


**Supplementary Figure 4.** Forest plot and funnel plot mean difference with fixed-effect models of RVEF (%) for VT development in rTOF. M-H: Mantel Haenszel. CI: Confidence interval.


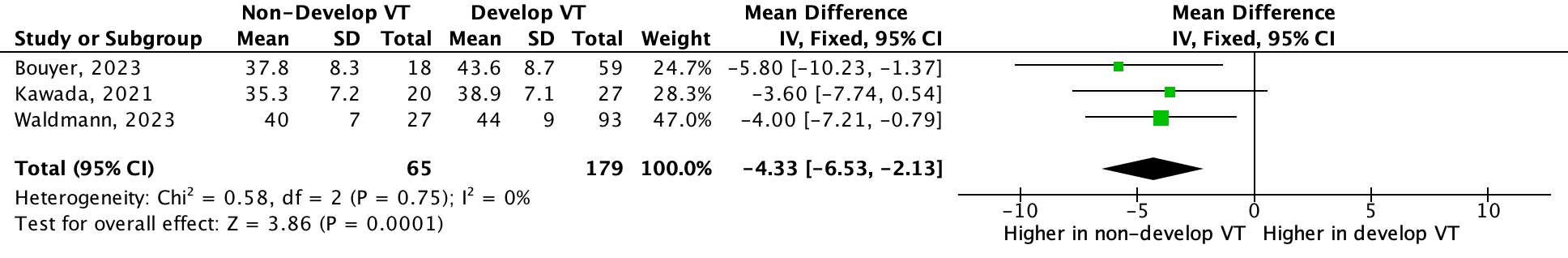


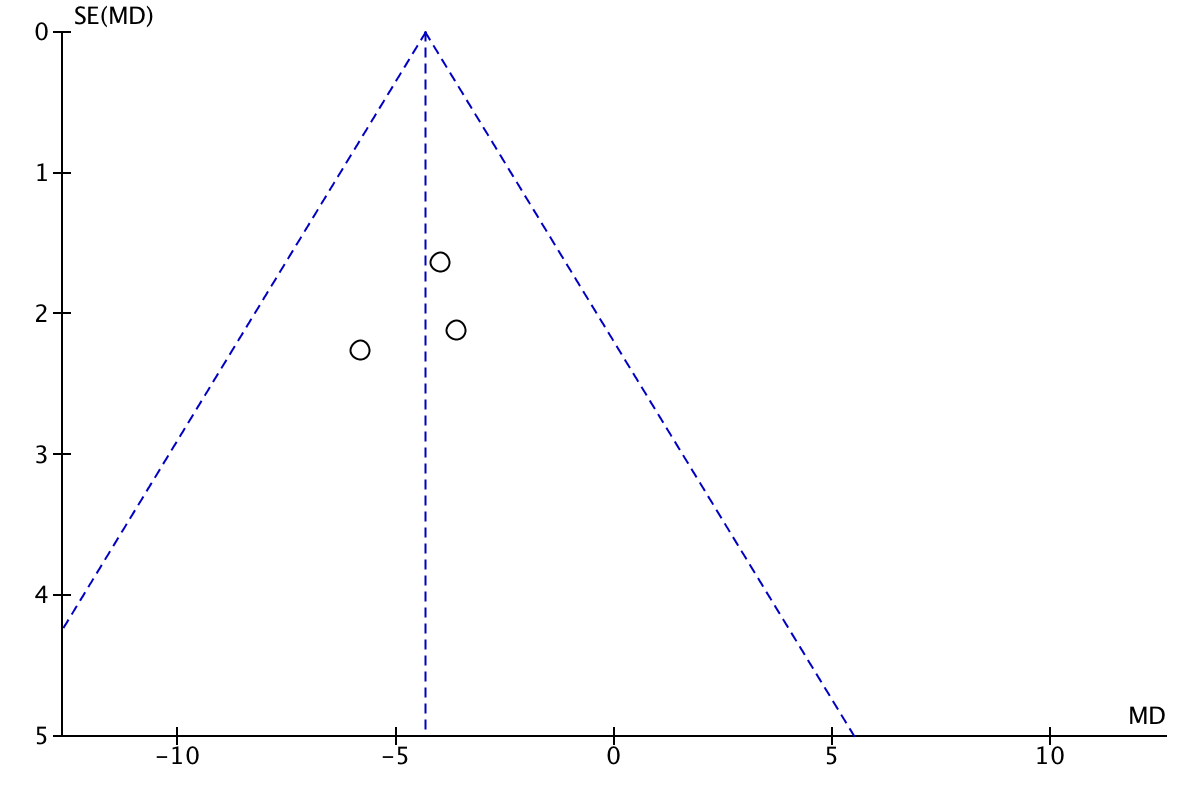


**Supplementary Figure 5.** Forest plot and funnel plot mean difference with random-effect models of RVEDV index for VT development in rTOF. M-H: Mantel Haenszel. CI: Confidence interval.

**
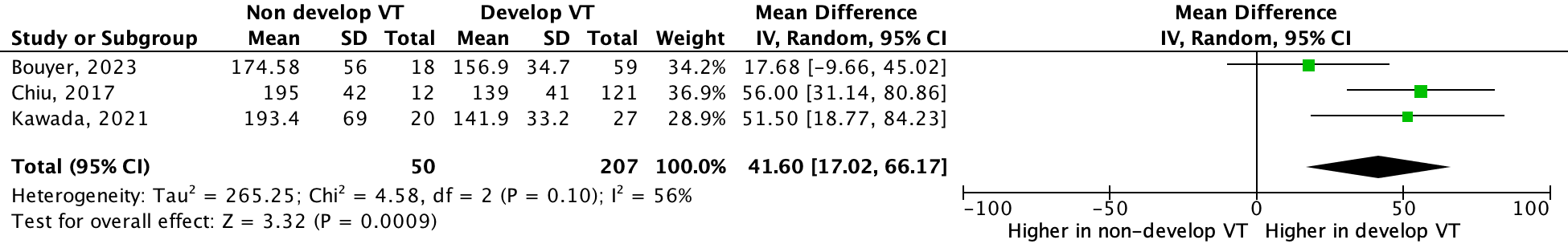
**

**
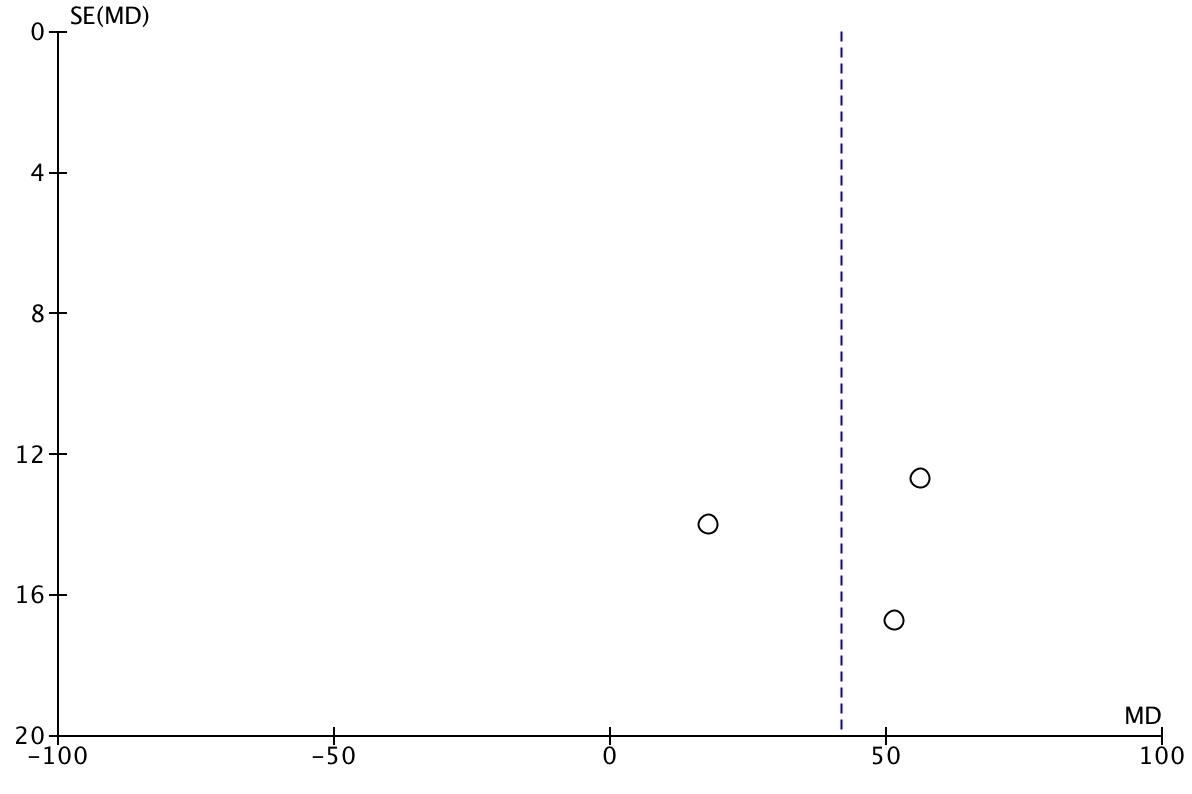
**

**Supplementary Figure 6.** Forest plot and funnel plot risk ratio with random-effect models of age for VT development in rTOF. M-H: Mantel Haenszel. CI: Confidence interval. SE(log[RR]): Standard error (log[Risk ratio]).

**
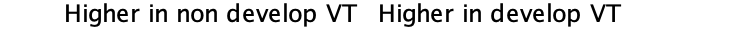
**
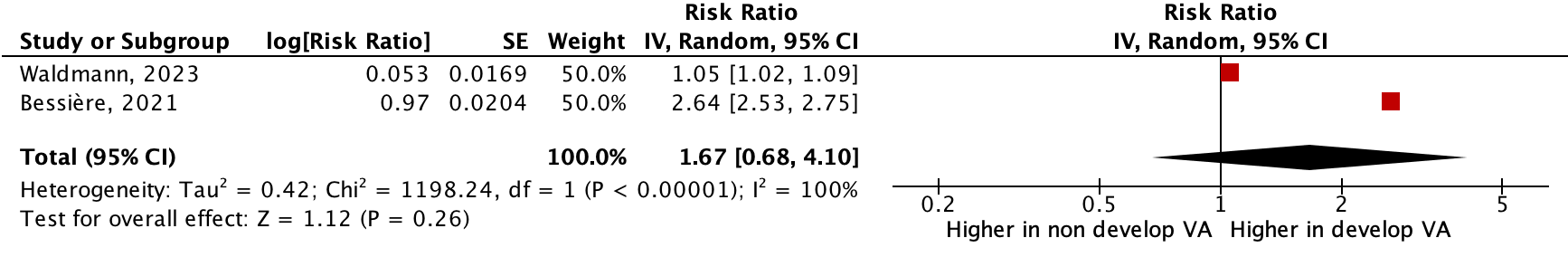


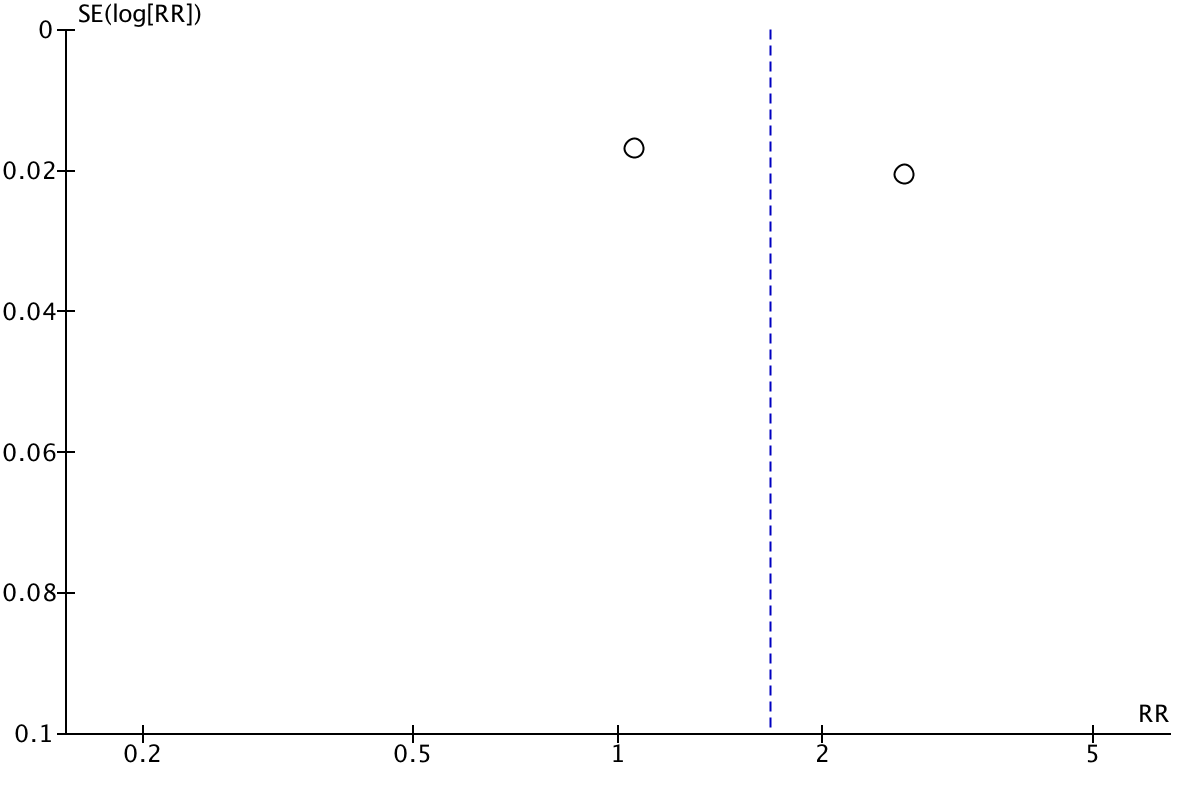


Forest plot and funnel plot reporting the MH RR of age for VT development in rTOF. Diamond indicates overall summary estimate for the analysis (width of the diamond represents the 95% CI); width of the shaded square, size of the population. CI, Confidence interval; MH, Mantel–Haenszel. random-effect model.

**Supplementary Figure 7.** Forest plot and funnel plot risk ratio with fixed-effect models of male gender for VT development in rTOF. M-H: Mantel Haenszel. CI: Confidence interval. SE(log[RR]): Standard error (log[Risk ratio]).

**
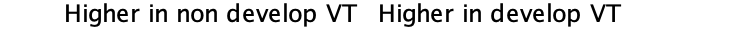
**
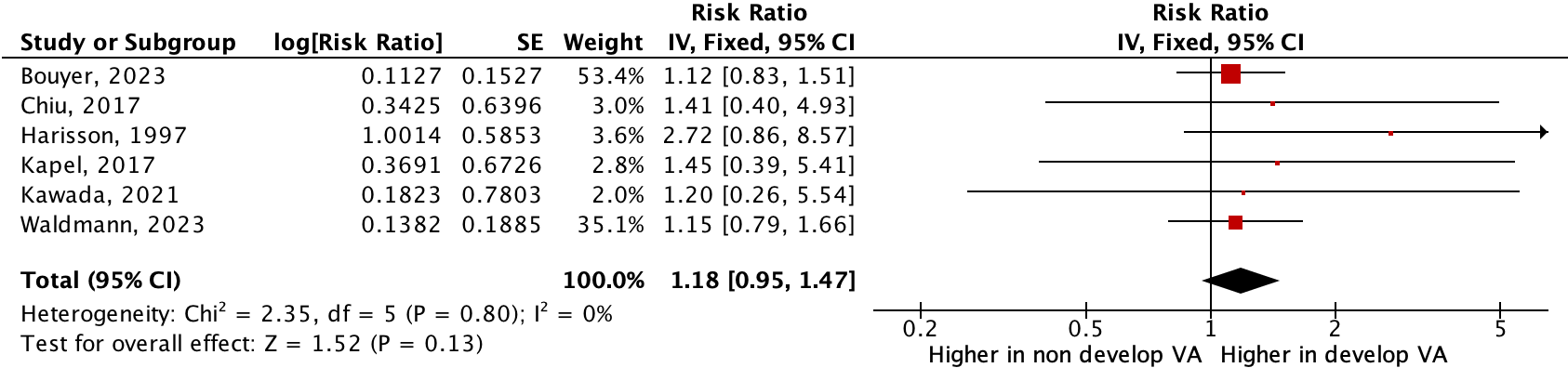


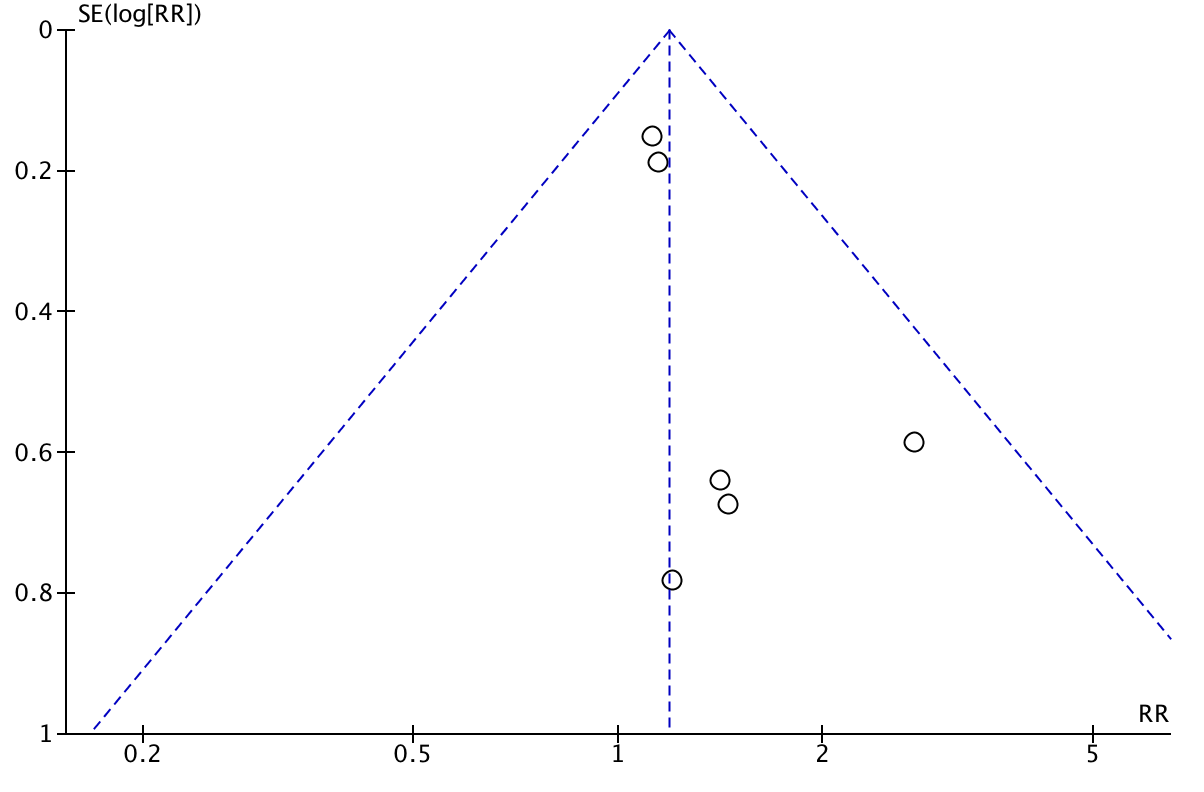


Forest plot and funnel plot reporting the MH RR of male gender for VT development in rTOF. Diamond indicates overall summary estimate for the analysis (width of the diamond represents the 95% CI); width of the shaded square, size of the population. CI, Confidence interval; MH, Mantel–Haenszel. Fixed-effect model.

**Supplementary Figure 8.** Forest plot and funnel plot risk ratio with fixed-effect models of age of TOF repair for VT development in rTOF. M-H: Mantel Haenszel. CI: Confidence interval. SE(log[RR]): Standard error (log[Risk ratio]).

**
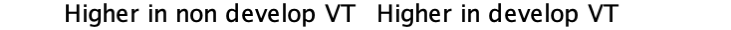

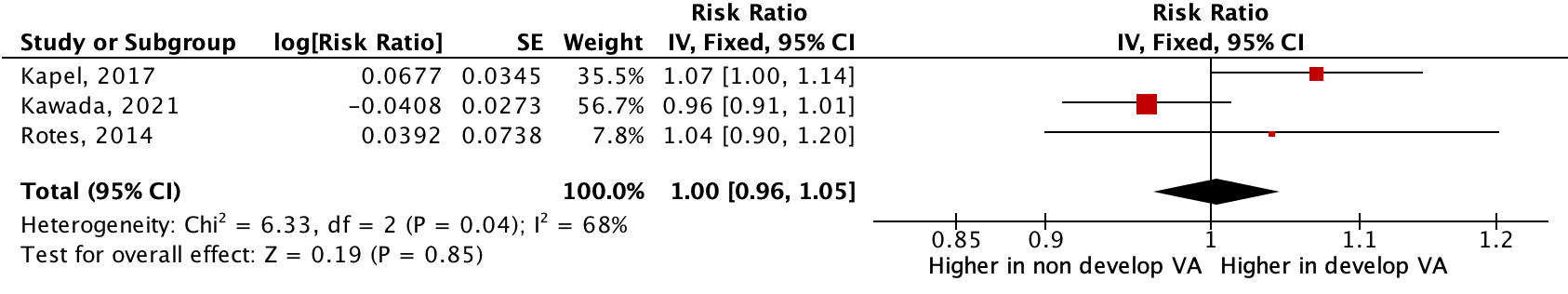
**


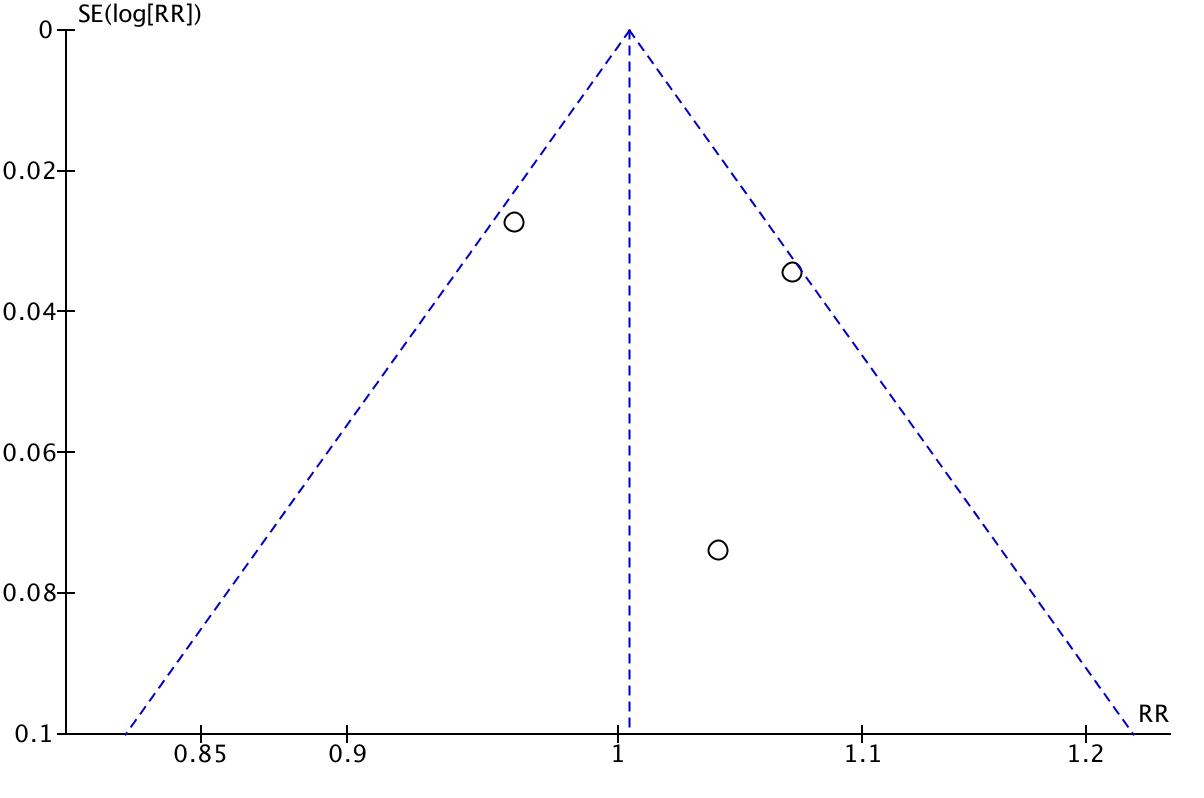
Forest plot and funnel plot reporting the MH RR of age of TOF repair for VT development in rTOF. Diamond indicates overall summary estimate for the analysis (width of the diamond represents the 95% CI); width of the shaded square, size of the population. CI, Confidence interval; MH, Mantel–Haenszel. fixed-effect model.

**Supplementary Figure 9.** Forest plot and funnel plot risk ratio with fixed-effect models of history of syncope for VT development in rTOF. M-H: Mantel Haenszel. CI: Confidence interval. SE(log[RR]): Standard error (log[Risk ratio]).

**
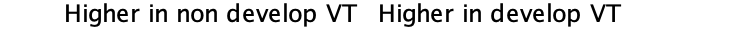
**
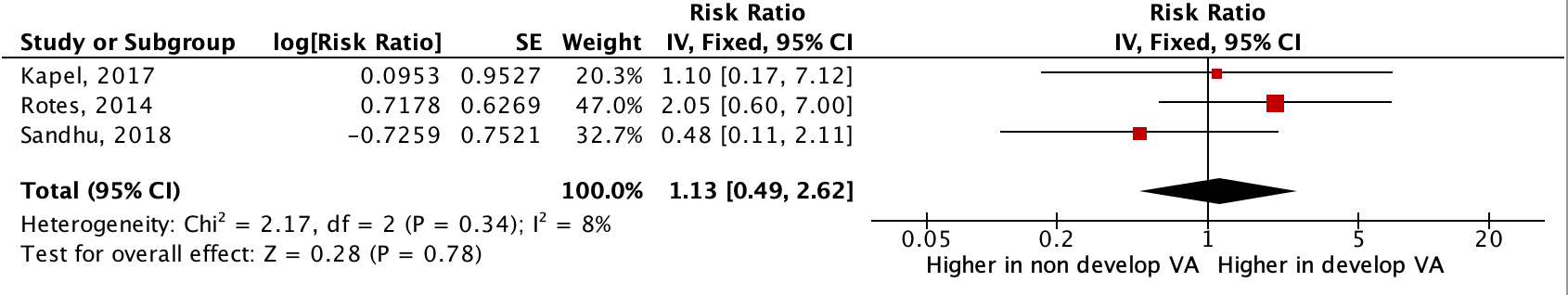


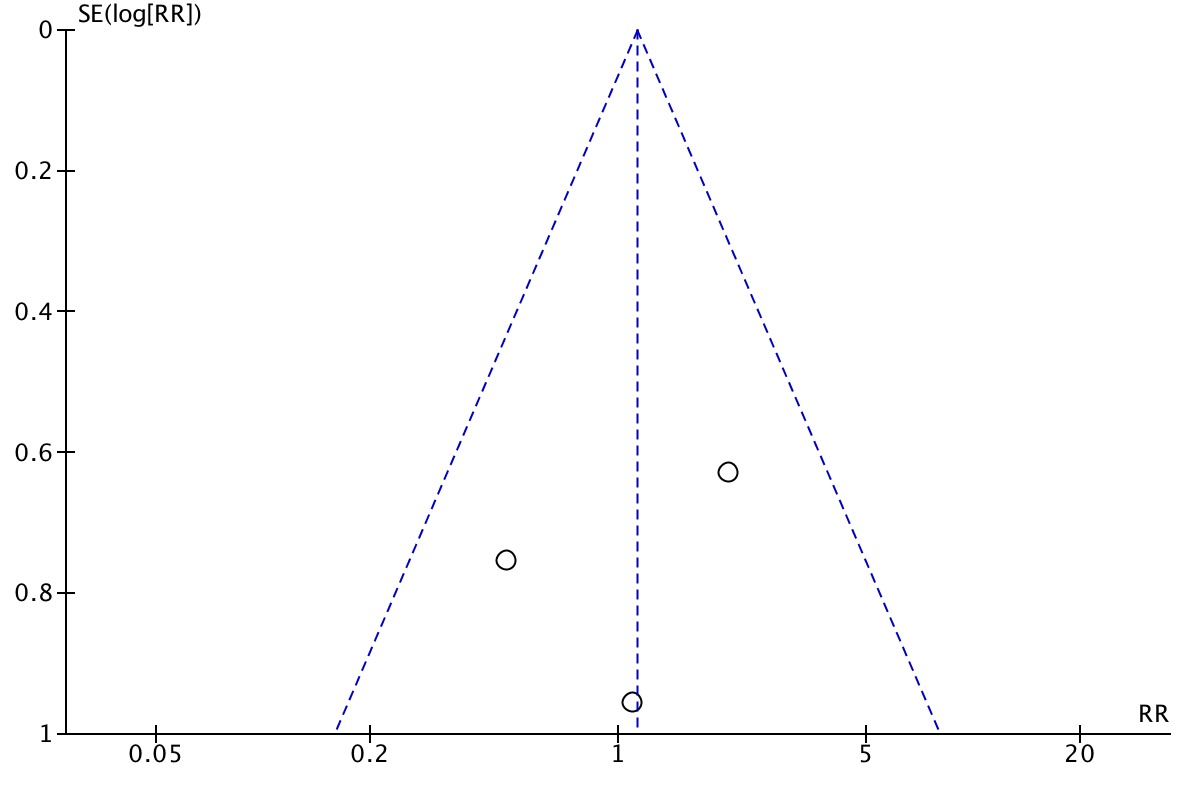


Forest plot and funnel plot reporting the MH RR of history of syncope for VT development in rTOF. Diamond indicates overall summary estimate for the analysis (width of the diamond represents the 95% CI); width of the shaded square, size of the population. CI, Confidence interval; MH, Mantel–Haenszel. fixed-effect model.

**Supplementary Figure 10.** Forest plot and funnel plot risk ratio with random-effect models of history of atrial arrhythmia for VT development in rTOF. M-H: Mantel Haenszel. CI: Confidence interval. SE(log[RR]): Standard error (log[Risk ratio]).

**
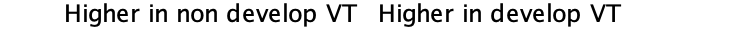
**
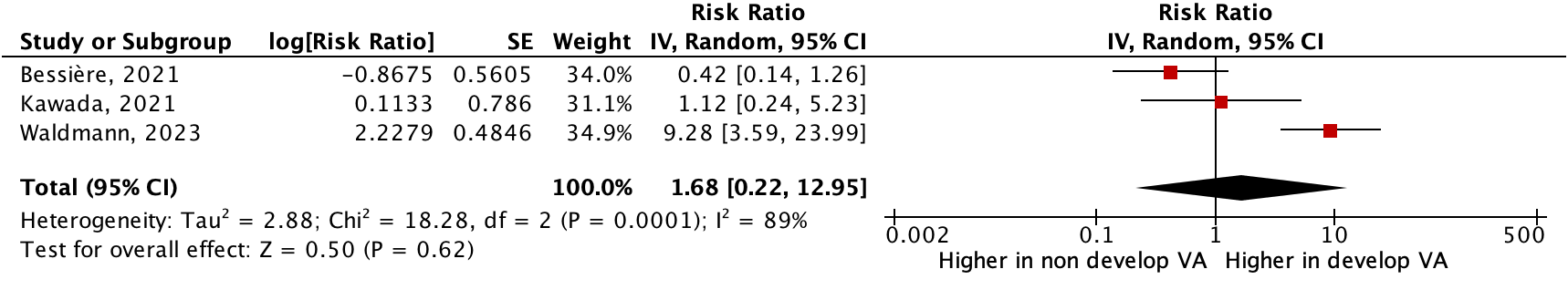


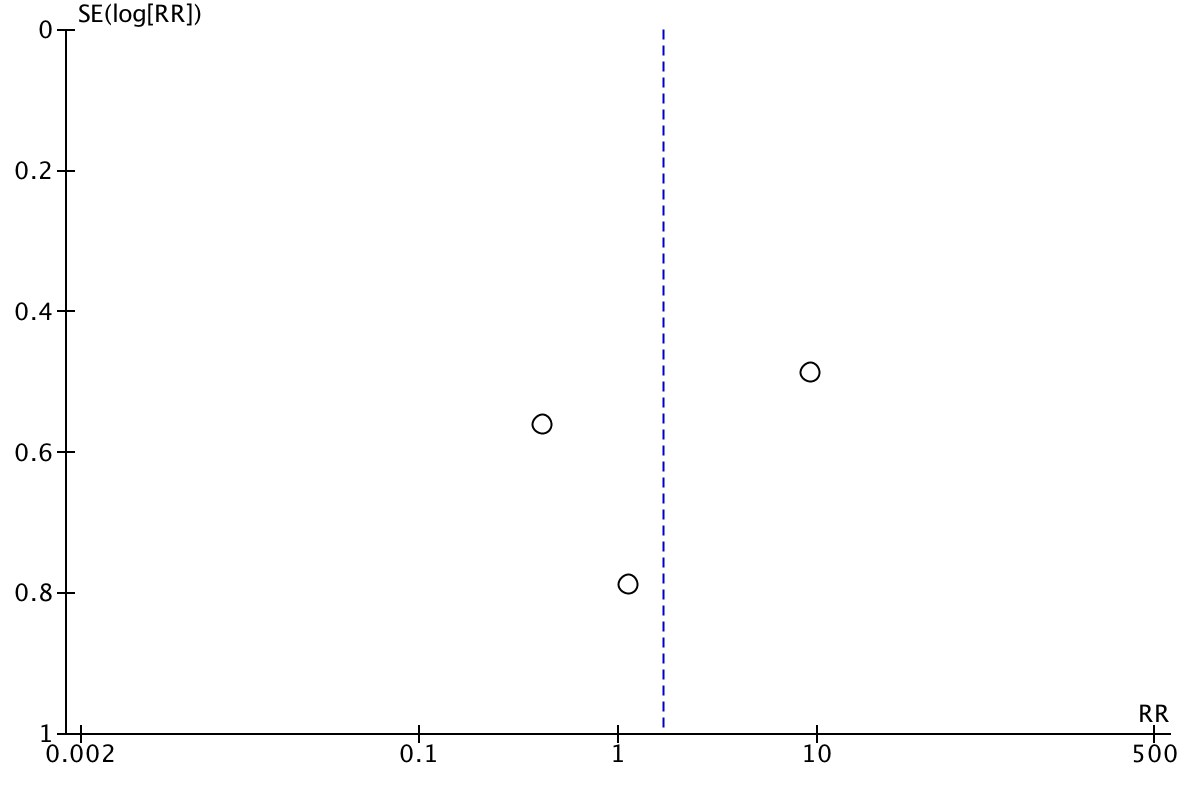


Forest plot and funnel plot reporting the MH RR of history of atrial arrhythmia for VT development in rTOF. Diamond indicates overall summary estimate for the analysis (width of the diamond represents the 95% CI); width of the shaded square, size of the population. CI, Confidence interval; MH, Mantel–Haenszel. random-effect model.

**Supplementary Figure 11.** Forest plot and funnel plot risk ratio with fixed-effect models of history of ventriculotomy/incision for VT development in rTOF. M-H: Mantel Haenszel. CI: Confidence interval. SE(log[RR]): Standard error (log[Risk ratio]).

**
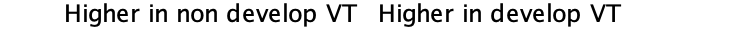
**
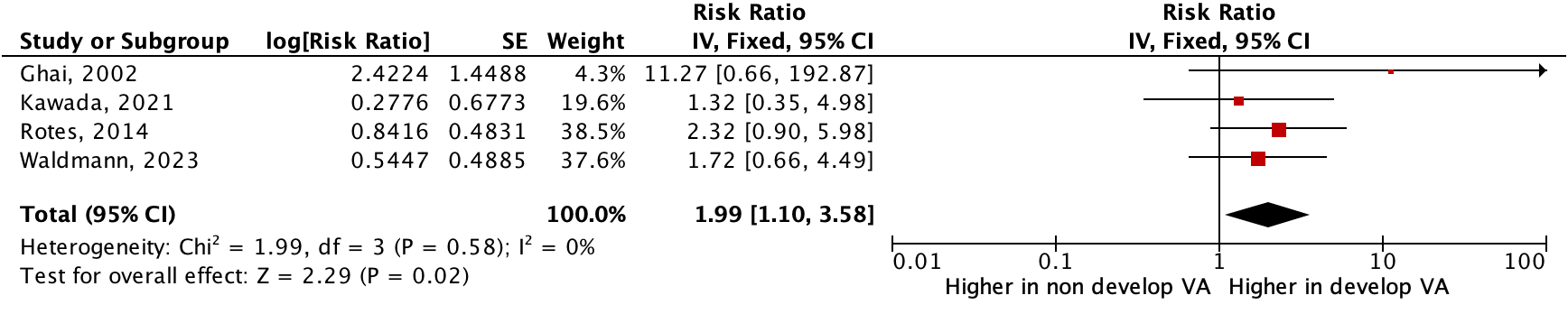


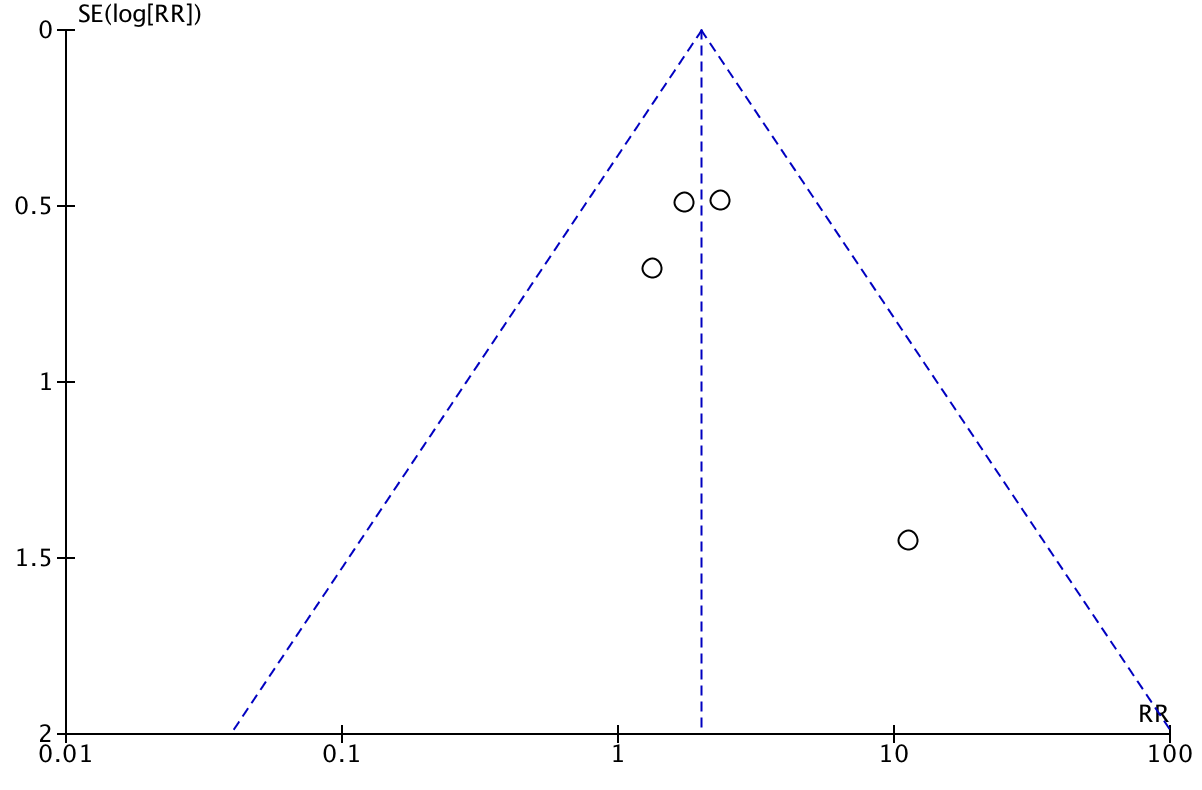


Forest plot and funnel plot reporting the MH RR of history of ventriculotomy/incision for VT development in rTOF. Diamond indicates overall summary estimate for the analysis (width of the diamond represents the 95% CI); width of the shaded square, size of the population. CI, Confidence interval; MH, Mantel–Haenszel. fixed-effect model.

**Supplementary Figure 12.** Forest plot and funnel plot risk ratio with fixed-effect models of transannular patch for VT development in rTOF. M-H: Mantel Haenszel. CI: Confidence interval. SE(log[RR]): Standard error (log[Risk ratio]).

**
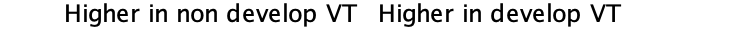

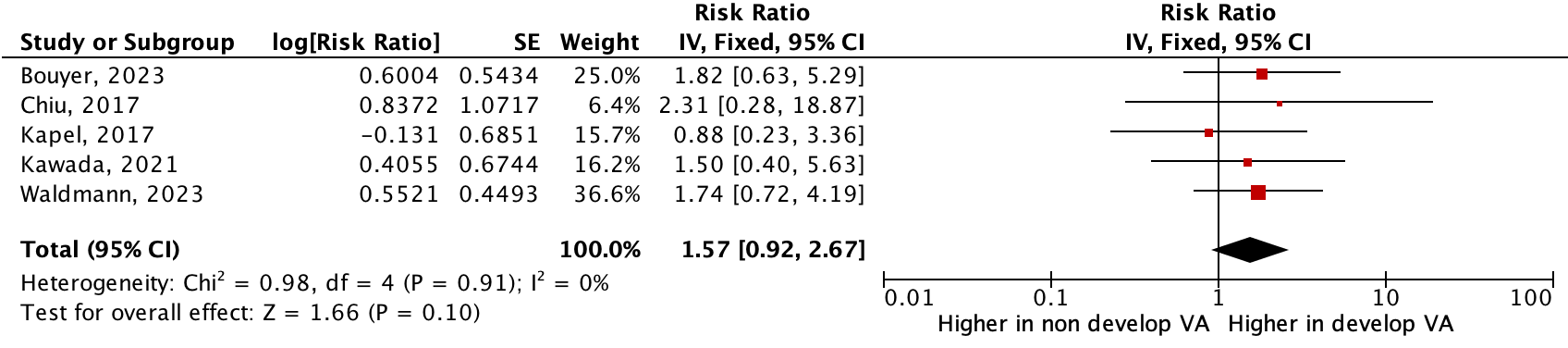
**

**
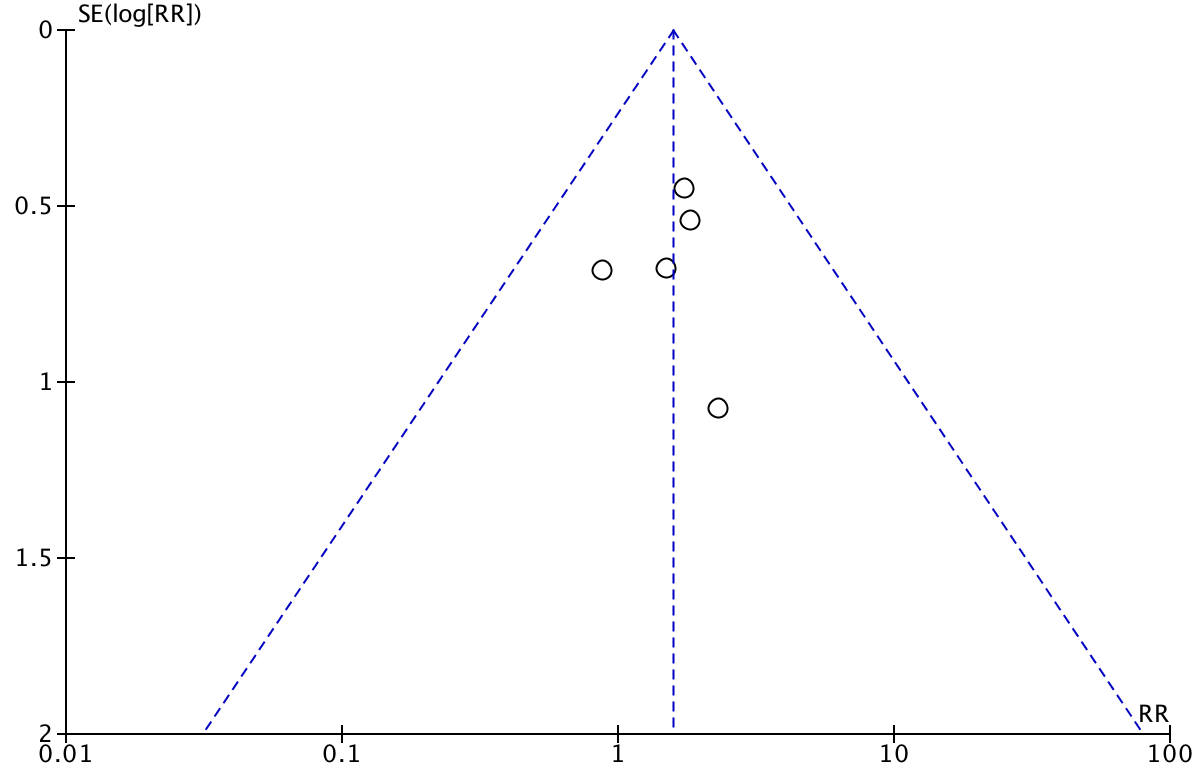
**

Forest plot and funnel plot reporting the MH RR of transannular patch for VT development in rTOF. Diamond indicates overall summary estimate for the analysis (width of the diamond represents the 95% CI); width of the shaded square, size of the population. CI, Confidence interval; MH, Mantel–Haenszel. fixed-effect model.

**Supplementary Figure 13.** Forest plot and funnel plot risk ratio with random-effect models of prior pulmonary valve repair for VT development in rTOF. M-H: Mantel Haenszel. CI: Confidence interval. SE(log[RR]): Standard error (log[Risk ratio]).

**
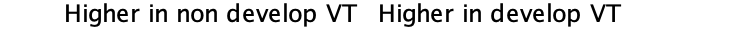

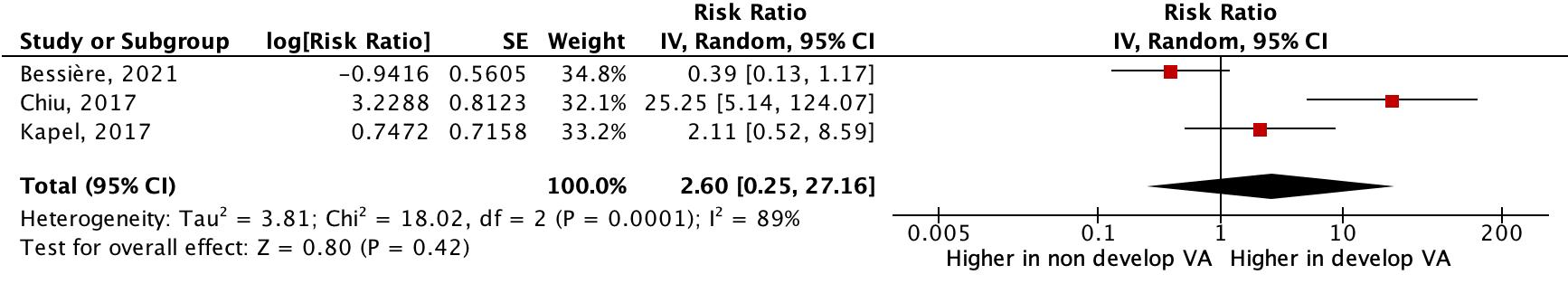
**

**
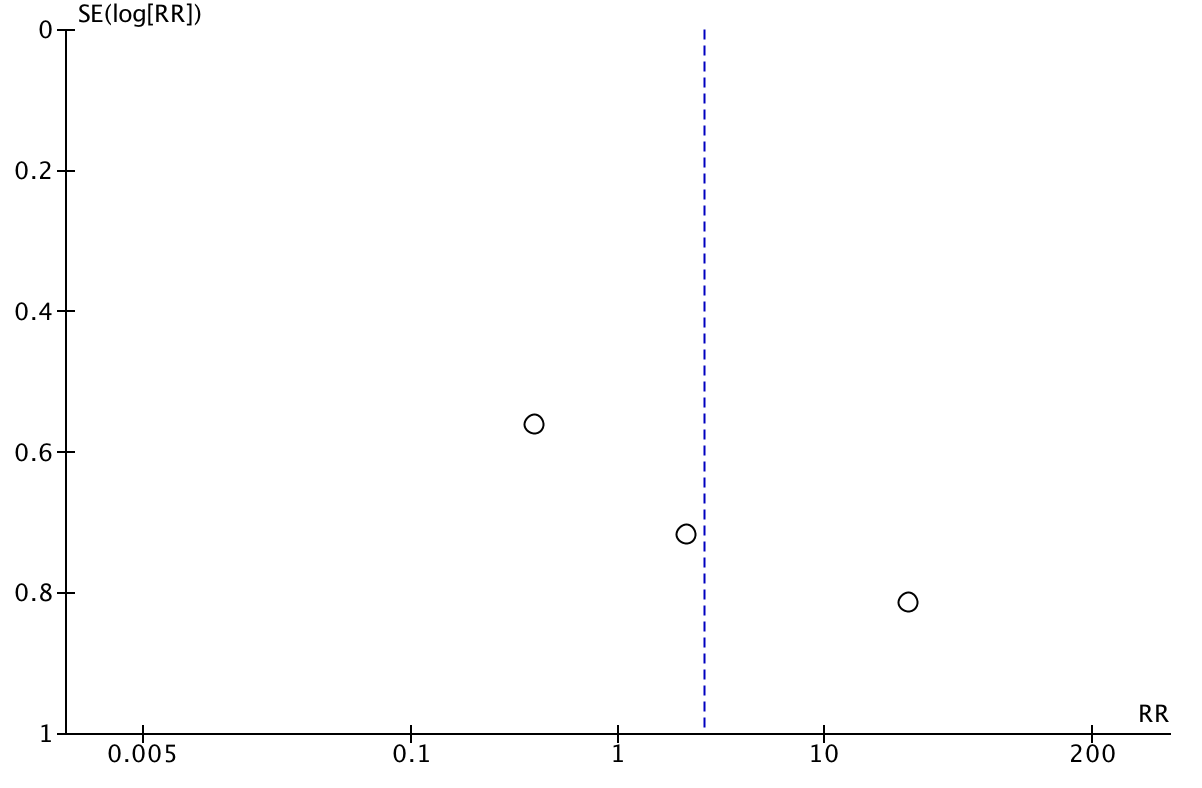
**

Forest plot and funnel plot reporting the MH RR of pulmonary valve repair for VT development in rTOF. Diamond indicates overall summary estimate for the analysis (width of the diamond represents the 95% CI); width of the shaded square, size of the population. CI, Confidence interval; MH, Mantel–Haenszel. random-effect model.

**Supplementary Figure 14.** Forest plot and funnel plot risk ratio with random-effect models of prior paloative shunt for VT development in rTOF. M-H: Mantel Haenszel. CI: Confidence interval. SE(log[RR]): Standard error (log[Risk ratio]).

**
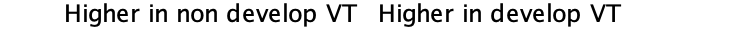

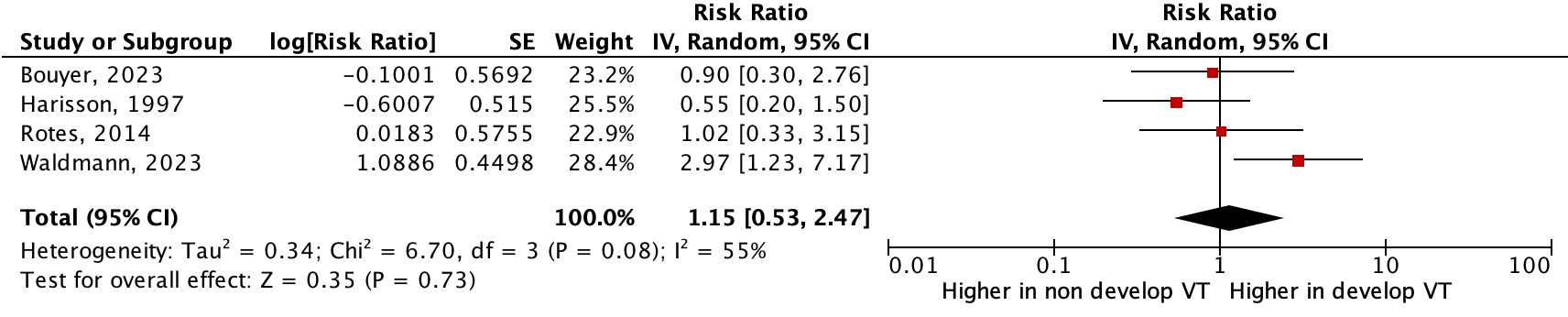
**

**
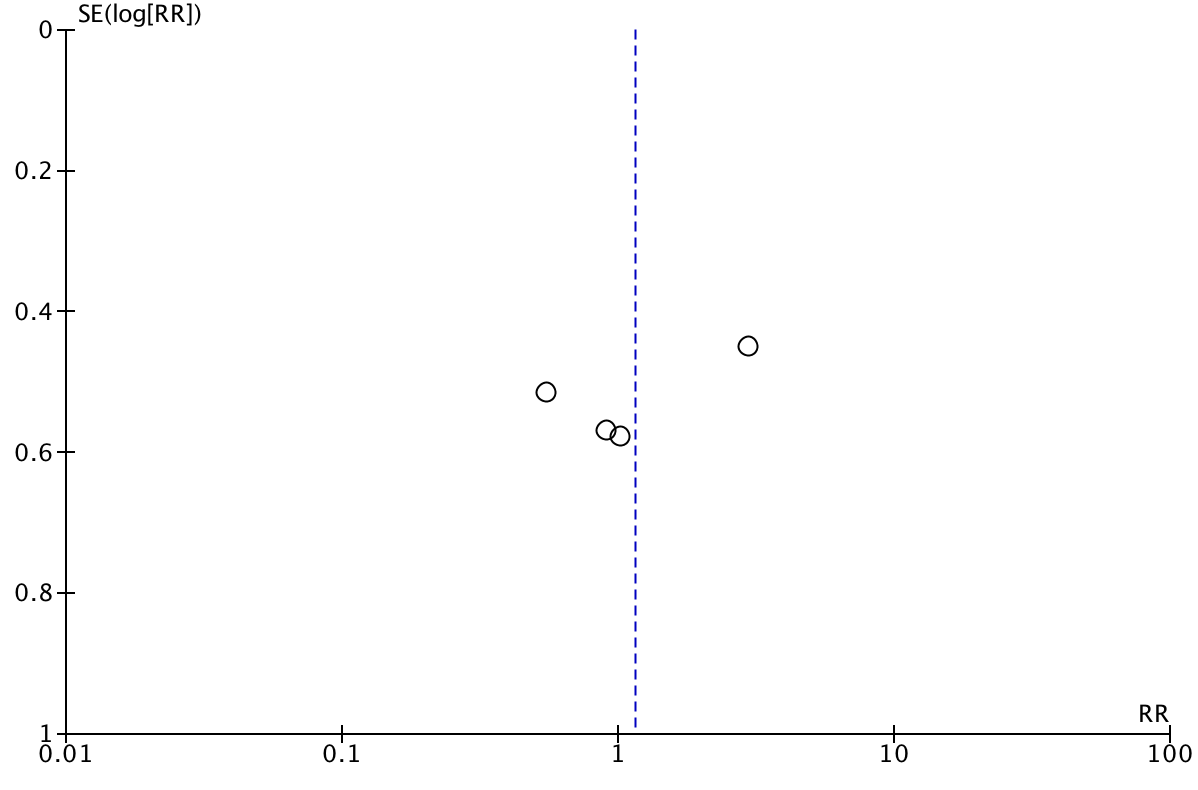
**

Forest plot and funnel plot reporting the MH RR of QRS duration >180ms for VT development in rTOF. Diamond indicates overall summary estimate for the analysis (width of the diamond represents the 95% CI); width of the shaded square, size of the population. CI, Confidence interval; MH, Mantel–Haenszel. random-effect model.

**Supplementary Figure 15.** Forest plot and funnel plot risk ratio with fixed-effect models of QRS duration > 180ms for VT development in rTOF. M-H: Mantel Haenszel. CI: Confidence interval. SE(log[RR]): Standard error (log[Risk ratio]).

**
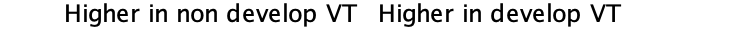

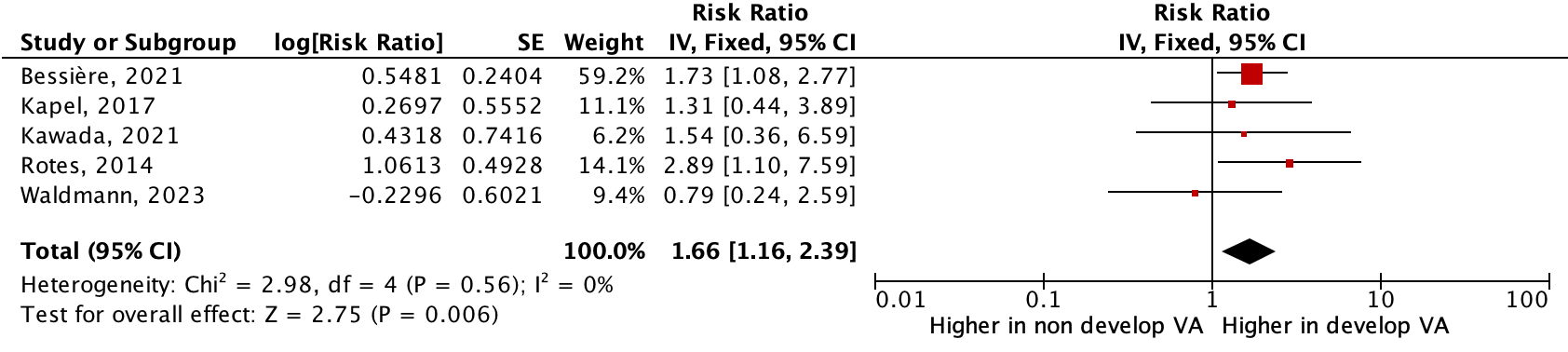
**


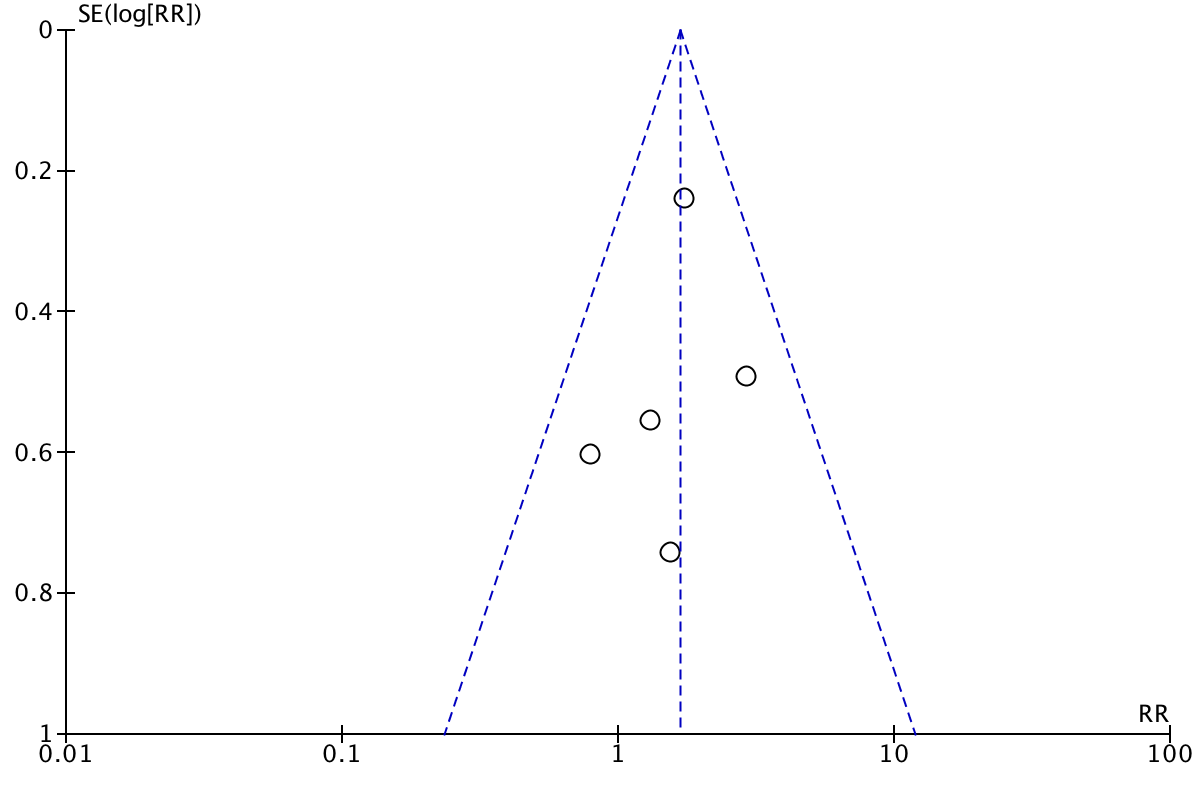
Forest plot and funnel plot reporting the MH RR of QRS duration > 180ms for VT development in rTOF. Diamond indicates overall summary estimate for the analysis (width of the diamond represents the 95% CI); width of the shaded square, size of the population. CI, Confidence interval; MH, Mantel–Haenszel. fixed-effect model.

**Supplementary Figure 16.** Forest plot and funnel plot risk ratio with fixed-effect models of fragmented QRS for VT development in rTOF. M-H: Mantel Haenszel. CI: Confidence interval. SE(log[RR]): Standard error (log[Risk ratio]).

**
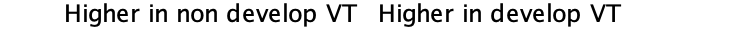

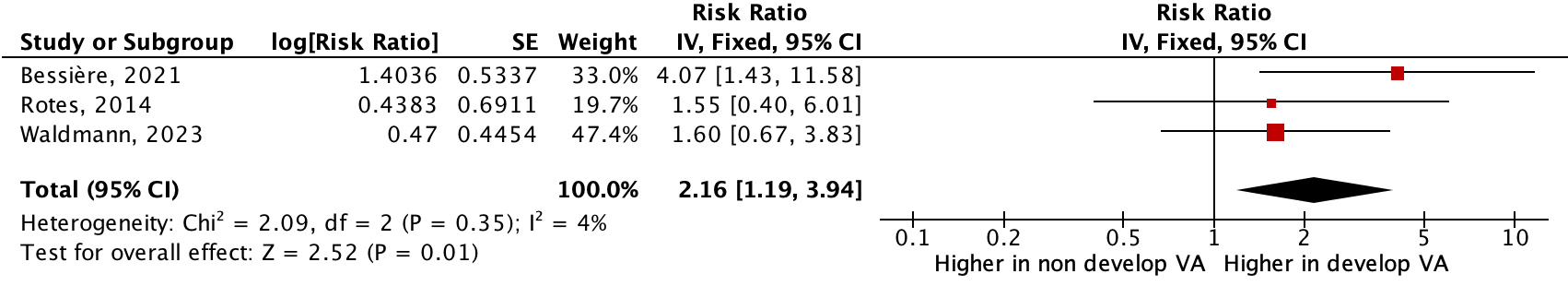
**

**
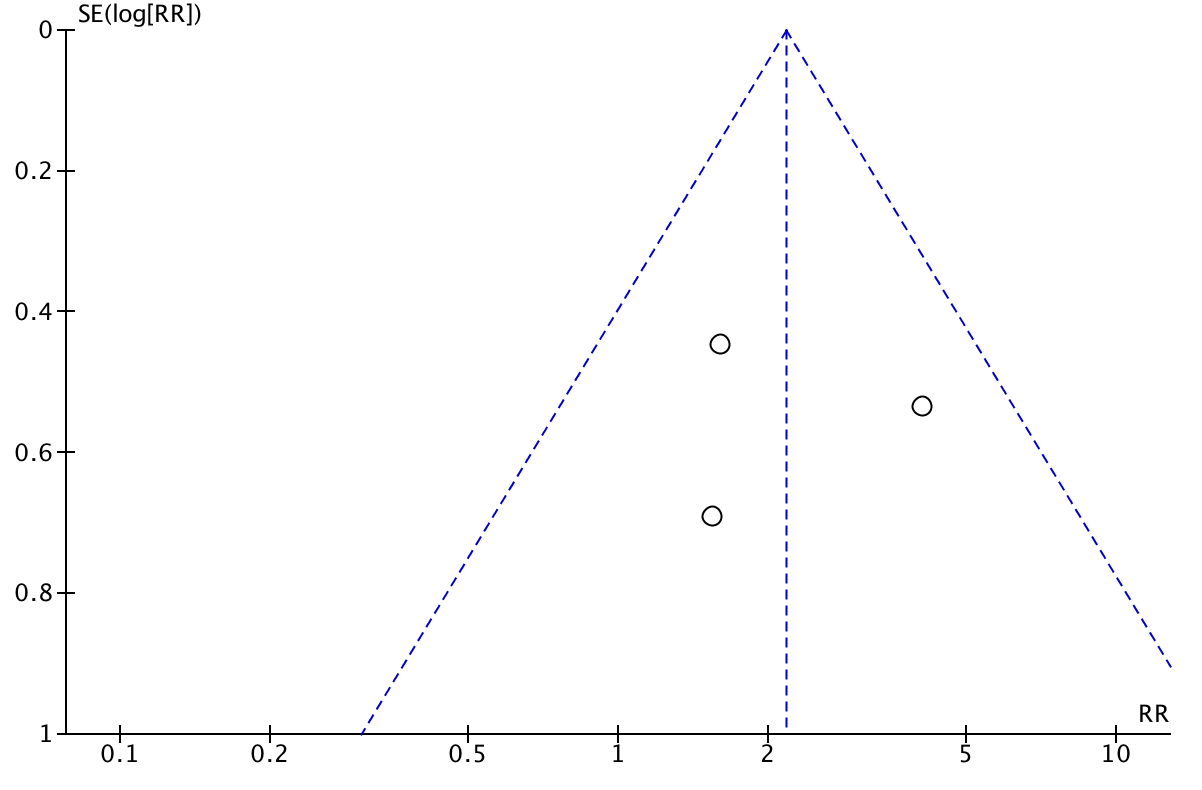
**

Forest plot and funnel plot reporting the MH RR of fragmented QRS for VT development in rTOF. Diamond indicates overall summary estimate for the analysis (width of the diamond represents the 95% CI); width of the shaded square, size of the population. CI, Confidence interval; MH, Mantel–Haenszel. fixed-effect model.

**Supplementary Figure 17.** Forest plot and funnel plot risk ratio with random-effect models of moderate to severe pulmonary regurgitation for VT development in rTOF. M-H: Mantel Haenszel. CI: Confidence interval. SE(log[RR]): Standard error (log[Risk ratio]).

**
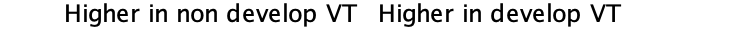

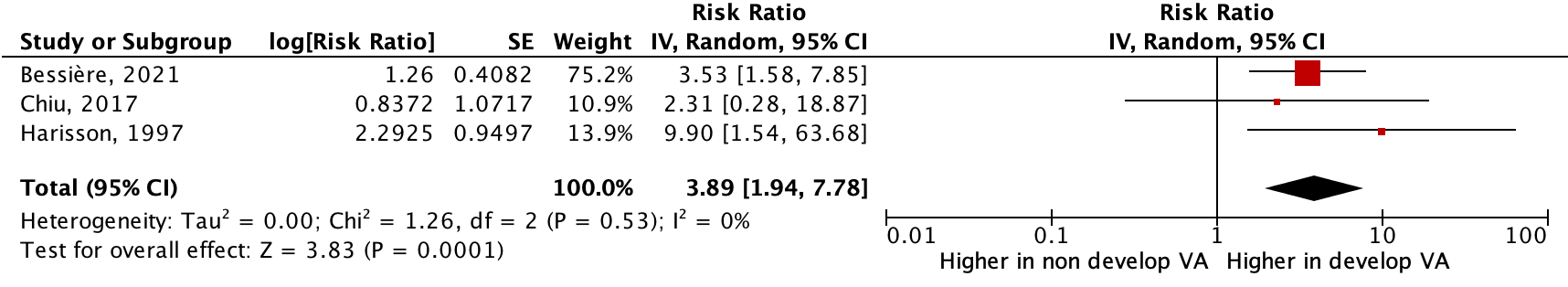
**

**
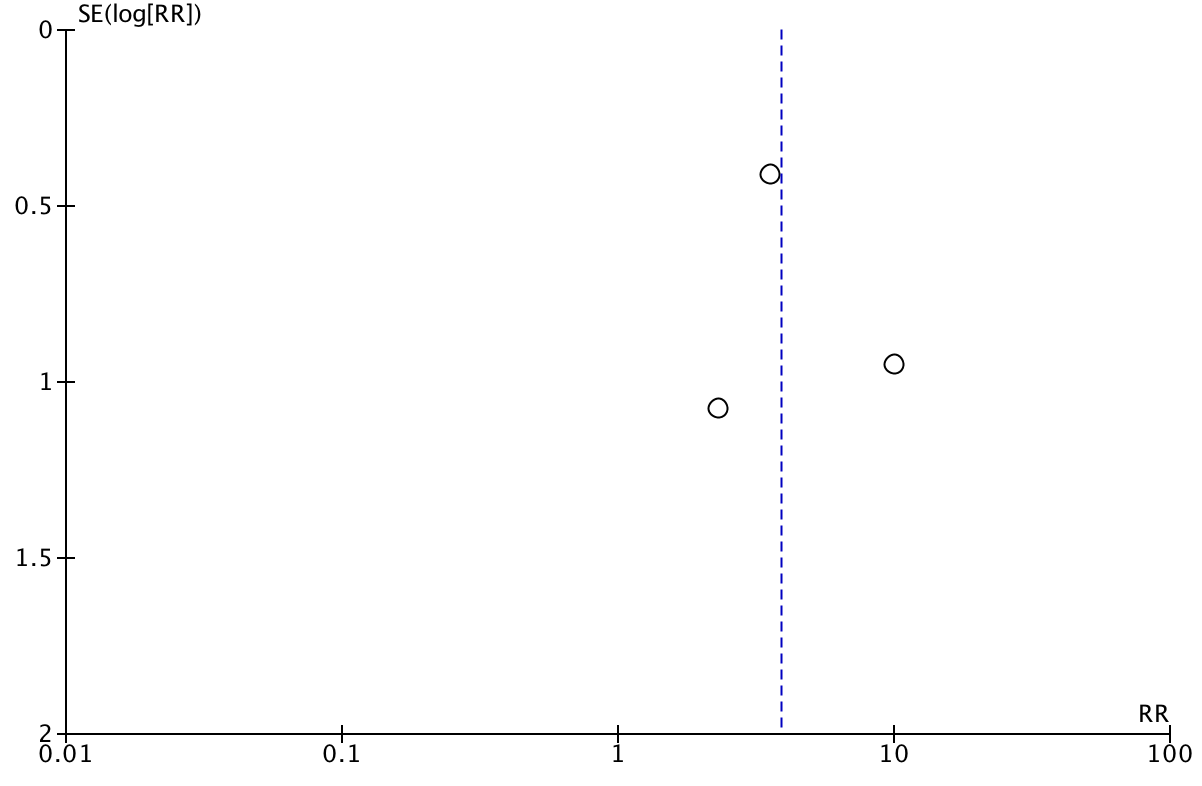
**

Forest plot and funnel plot reporting the MH RR of moderate to severe pulmonary regurgitation for VT development in rTOF. Diamond indicates overall summary estimate for the analysis (width of the diamond represents the 95% CI); width of the shaded square, size of the population. CI, Confidence interval; MH, Mantel–Haenszel. random-effect model.

**Supplementary Figure 18.** Forest plot and funnel plot risk ratio with random-effect models of moderate to severe right ventricular dysfunction for VT development in rTOF. M-H: Mantel Haenszel. CI: Confidence interval. SE(log[RR]): Standard error (log[Risk ratio]).

**
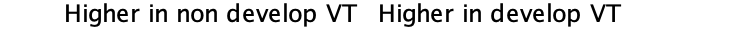

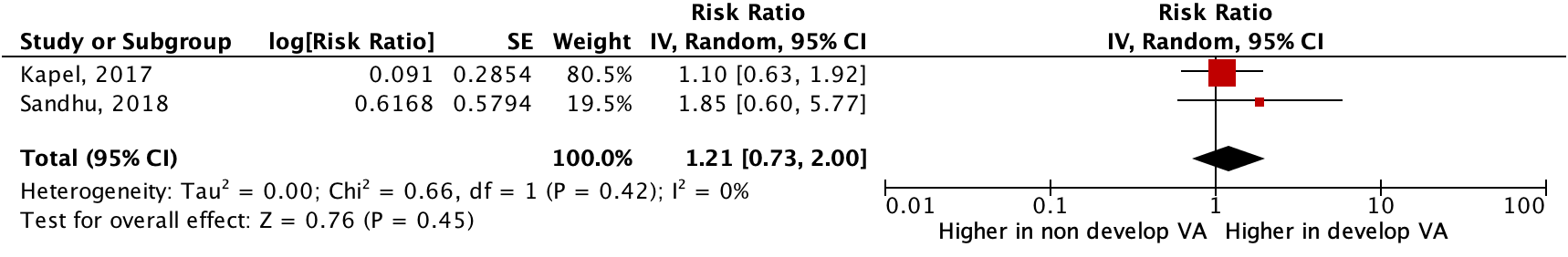
**

**
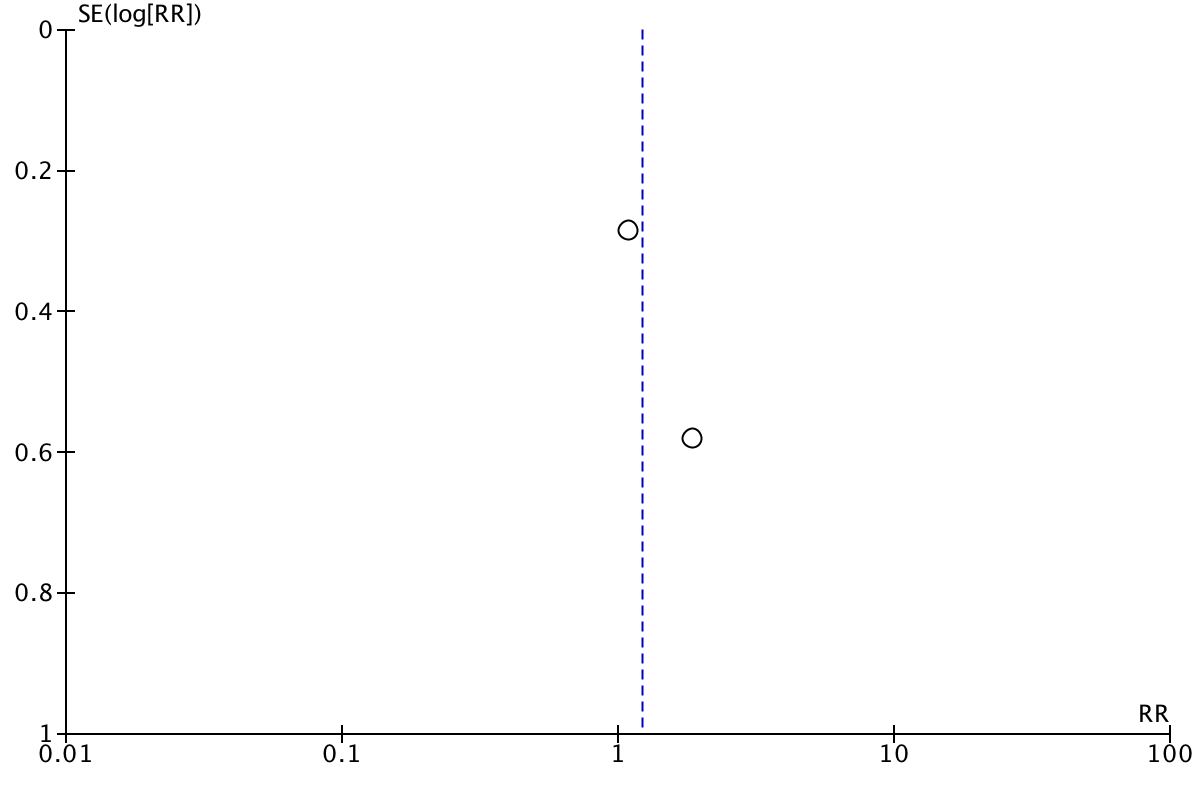
**

Forest plot and funnel plot reporting the MH RR of moderate to severe right ventricular dysfunction for VT development in rTOF. Diamond indicates overall summary estimate for the analysis (width of the diamond represents the 95% CI); width of the shaded square, size of the population. CI, Confidence interval; MH, Mantel–Haenszel. random-effect model.

**Supplementary Figure 19.** Forest plot and funnel plot risk ratio with random-effect models of left ventricular ejection fraction for VT development in rTOF. M-H: Mantel Haenszel. CI: Confidence interval. SE(log[RR]): Standard error (log[Risk ratio]).

**
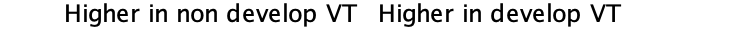

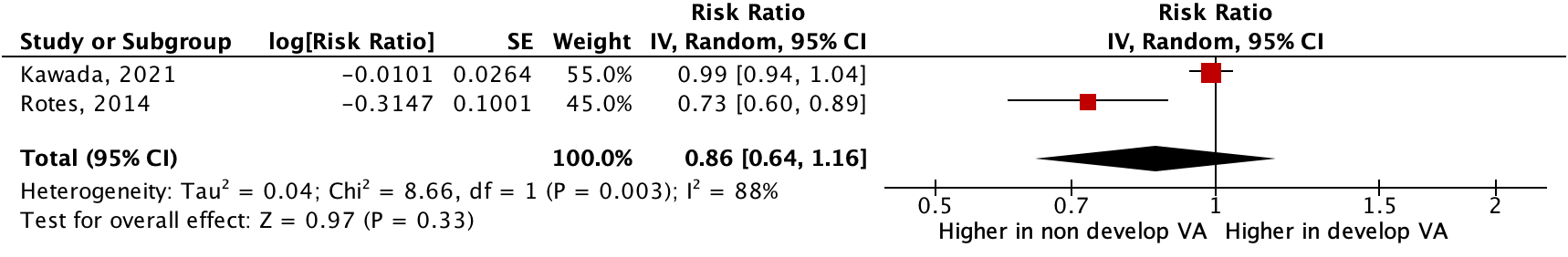
**

**
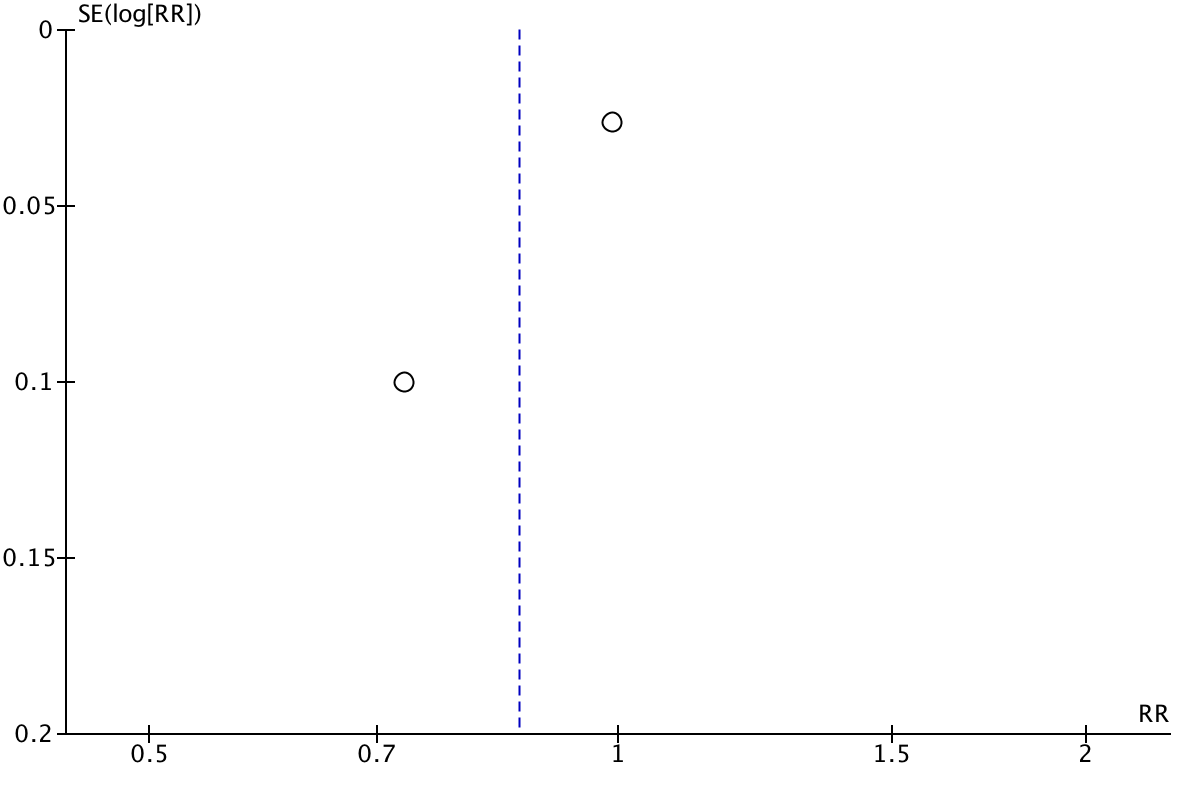
**

Forest plot and funnel plot reporting the MH RR of left ventricular ejection fraction for VT development in rTOF. Diamond indicates overall summary estimate for the analysis (width of the diamond represents the 95% CI); width of the shaded square, size of the population. CI, Confidence interval; MH, Mantel–Haenszel. random-effect model.

**Supplementary Figure 20.** Forest plot and funnel plot risk ratio with fixed-effect models of high PVC burden or NSVT on 24 hour Holter for VT development in rTOF. M-H: Mantel Haenszel. CI: Confidence interval. SE(log[RR]): Standard error (log[Risk ratio]).

**
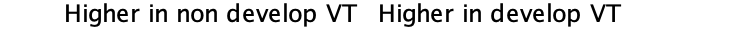

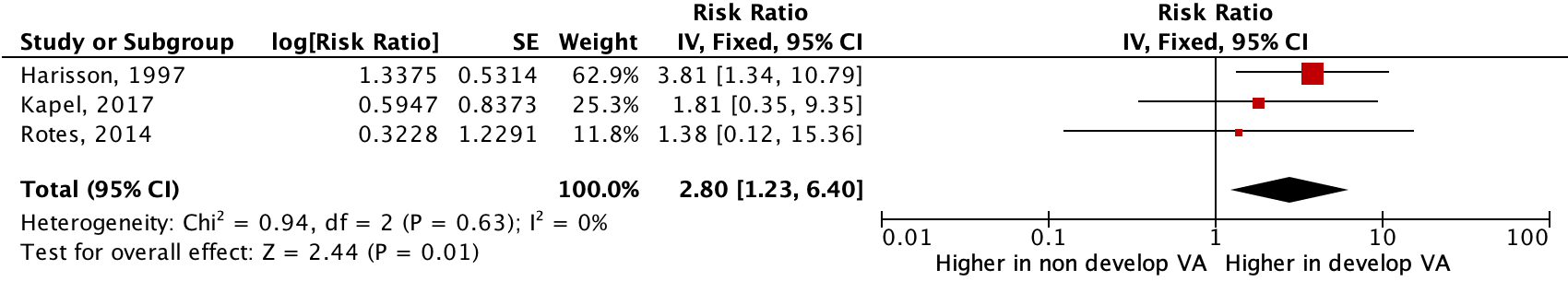
**


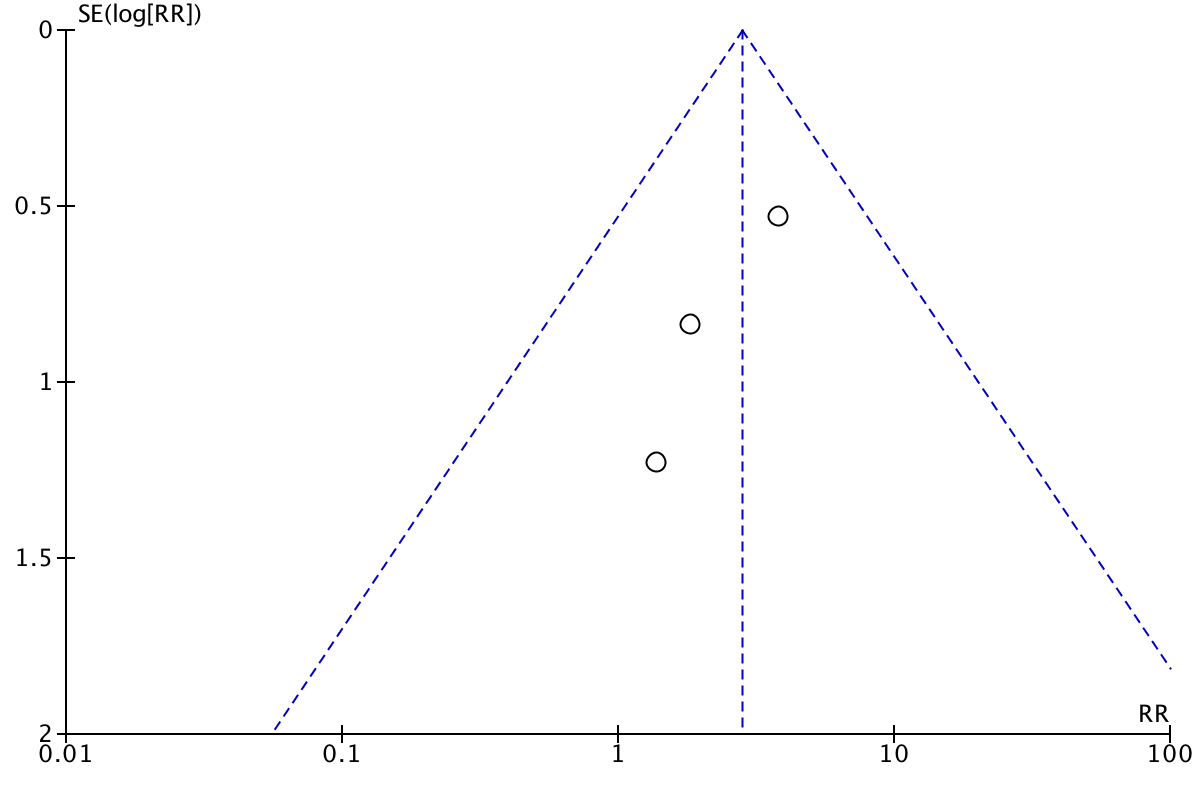
Forest plot and funnel plot reporting the MH RR of right ventricular dysfunction for VT development in rTOF. Diamond indicates overall summary estimate for the analysis (width of the diamond represents the 95% CI); width of the shaded square, size of the population. CI, Confidence interval; MH, Mantel–Haenszel. fixed-effect model.

**Supplementary Figure 21.** Forest plot and funnel plot risk ratio with random-effect models of late godolinum enchancement for VT development in rTOF. M-H: Mantel Haenszel. CI: Confidence interval. SE(log[RR]): Standard error (log[Risk ratio]).

**
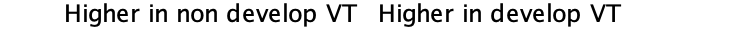

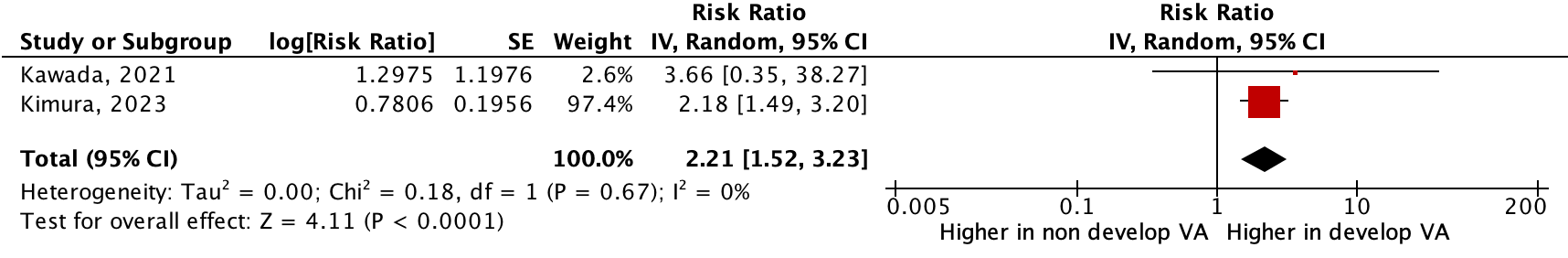
**

**
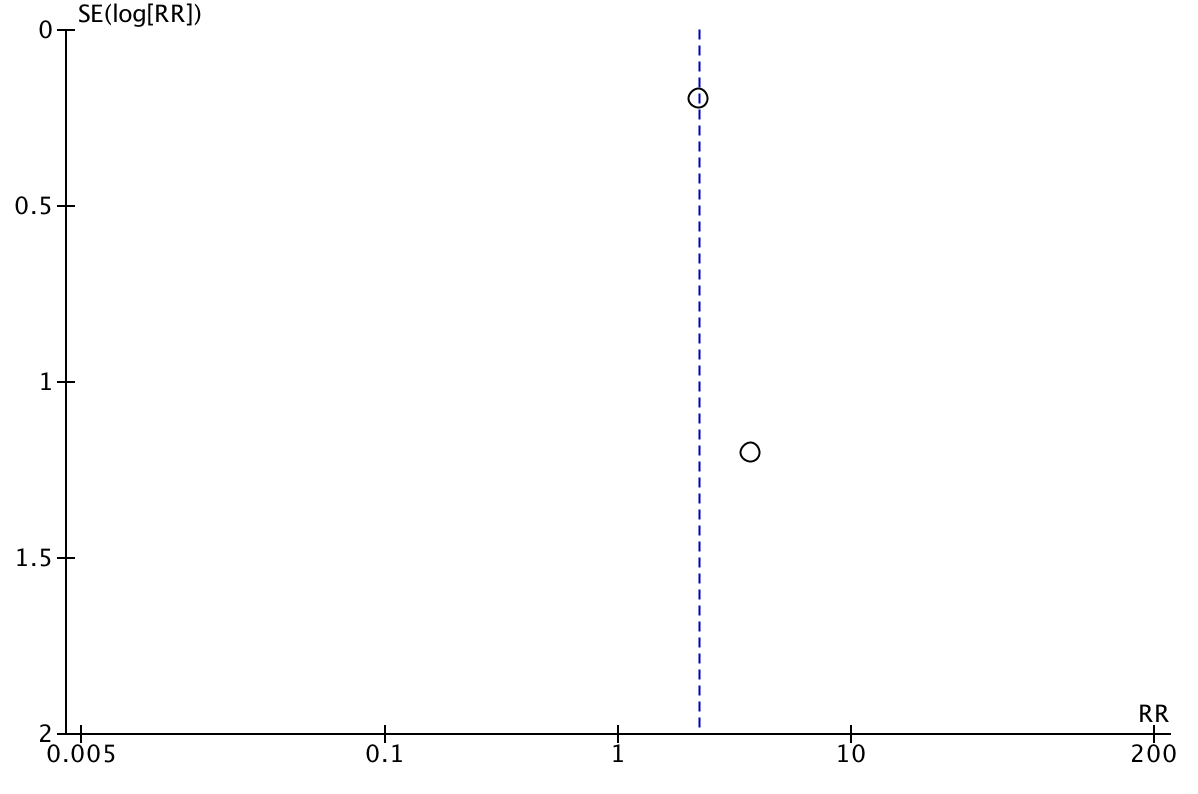
**

Forest plot and funnel plot reporting the MH RR of right ventricular late godolinum enchancement for VT development in rTOF. Diamond indicates overall summary estimate for the analysis (width of the diamond represents the 95% CI); width of the shaded square, size of the population. CI, Confidence interval; MH, Mantel–Haenszel. random-effect model.

**
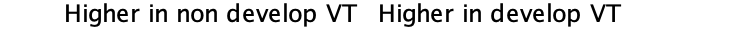
Supplementary Figure 22.** Forest plot and funnel plot risk ratio with random-effect models of programmed ventricular stimulation for VT development in rTOF. M-H: Mantel Haenszel. CI: Confidence interval. SE(log[RR]): Standard error (log[Risk ratio]).**
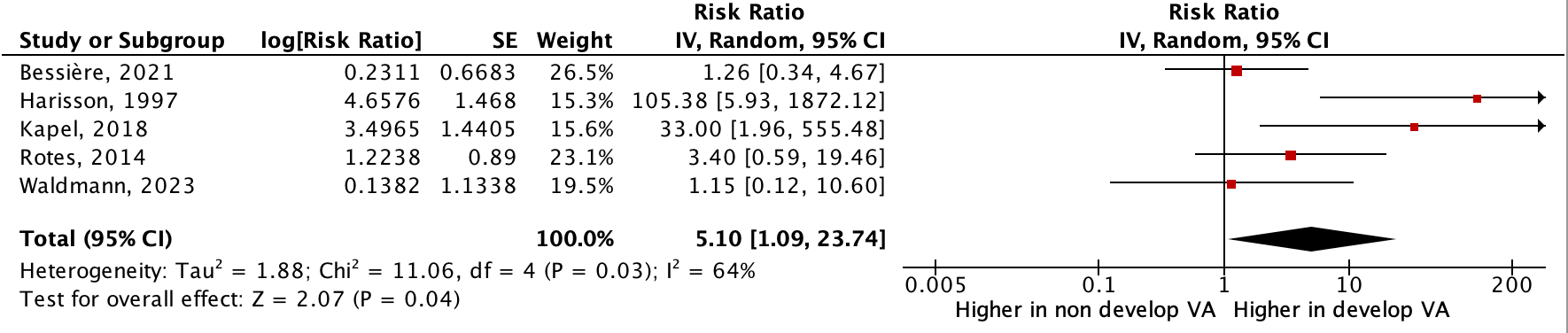
**

**
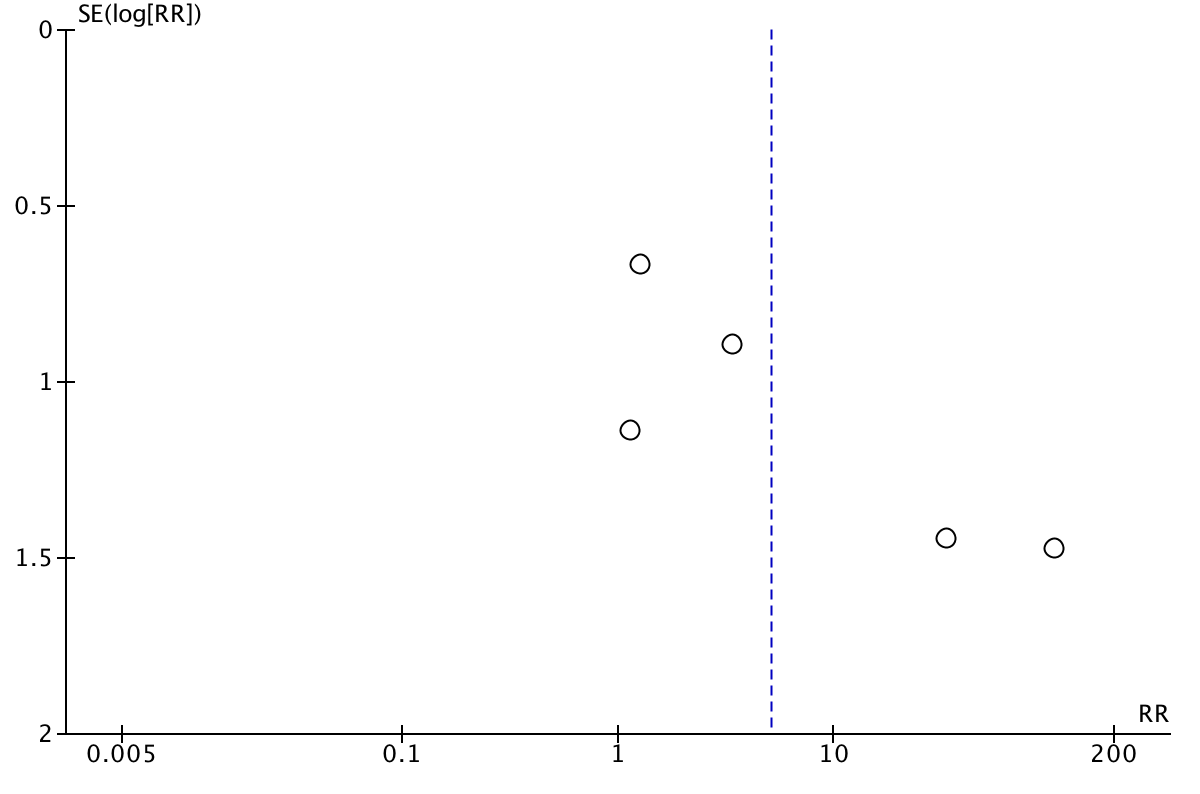
**

Forest plot and funnel plot reporting the MH RR of programmed ventricular stimulation for VT development in rTOF. Diamond indicates overall summary estimate for the analysis (width of the diamond represents the 95% CI); width of the shaded square, size of the population. CI, Confidence interval; MH, Mantel–Haenszel. random-effect model.

**Supplementary Figure 23.** Forest plot and funnel plot risk ratio with random-effect models of presence of slow conducting anatomical isthmus for VT development in rTOF. M-H: Mantel Haenszel. CI: Confidence interval. SE(log[RR]): Standard error (log[Risk ratio]).

**
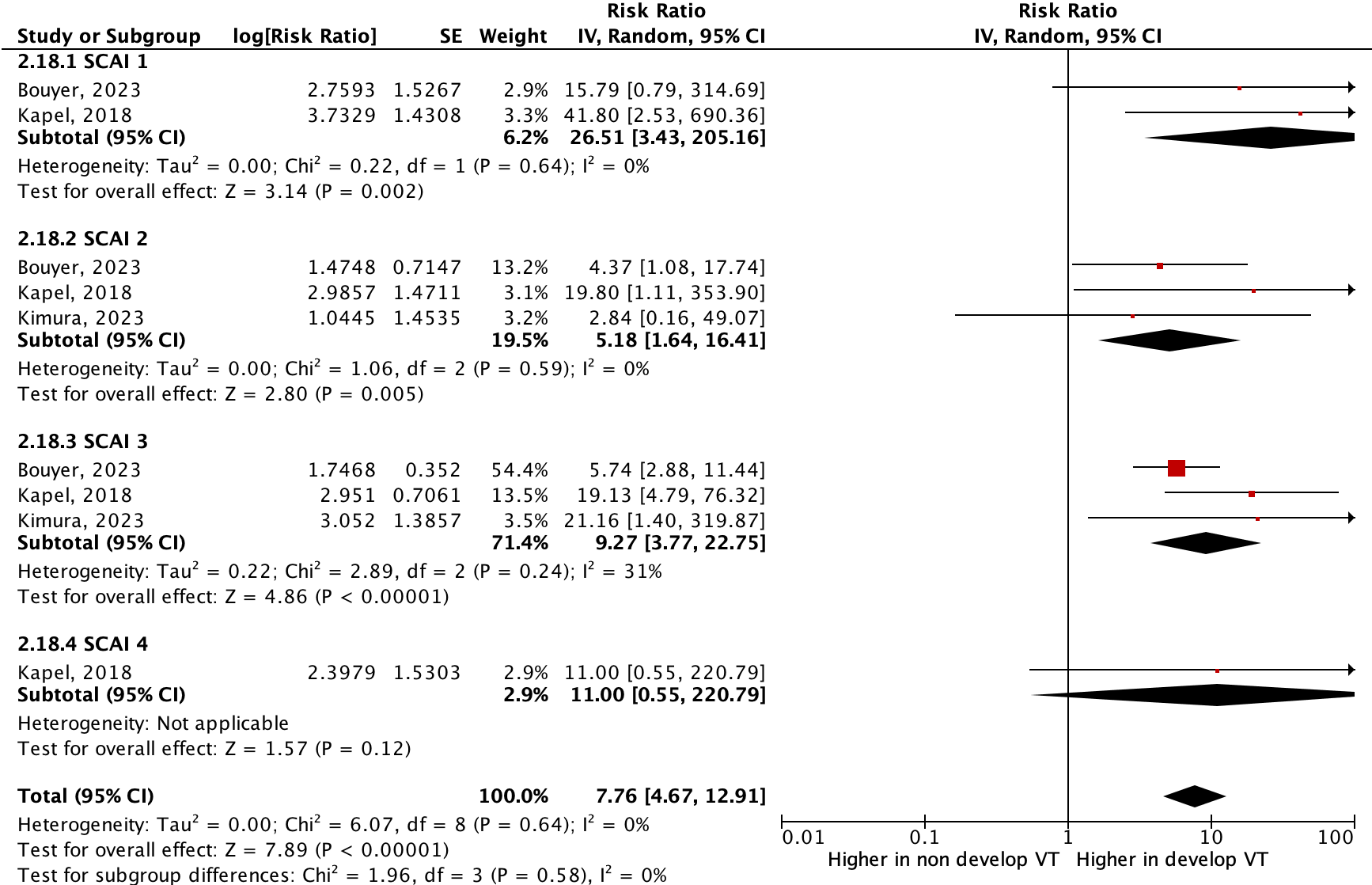
**

**
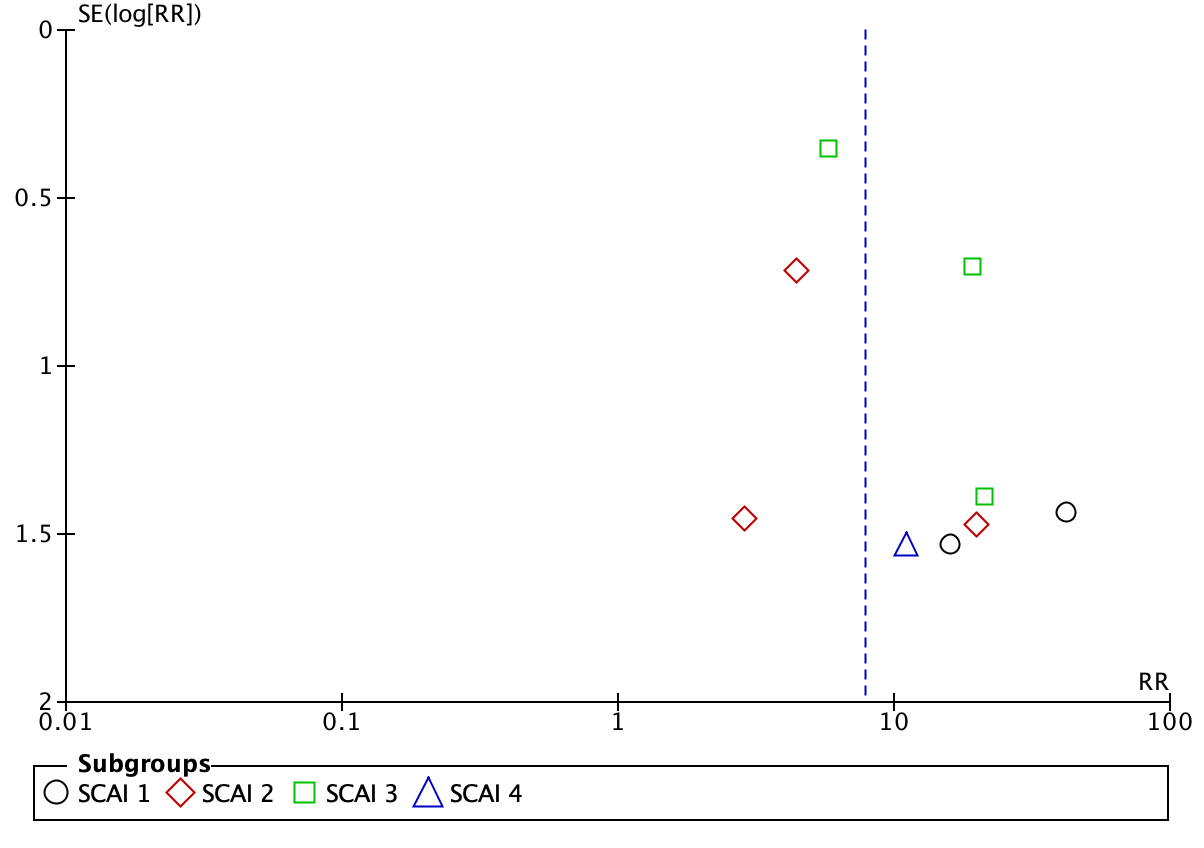
**

Forest plot and funnel plot reporting the MH RR of presence of slow conducting anatomical isthmus for VT development in rTOF. Diamond indicates overall summary estimate for the analysis (width of the diamond represents the 95% CI); width of the shaded square, size of the population. CI, Confidence interval; MH, Mantel–Haenszel. random-effect model.
